# Education necessity for veterinary-producer relationship creation and sustainability: a mixed method study

**DOI:** 10.3389/fvets.2025.1521440

**Published:** 2025-04-17

**Authors:** Nicola L. Ritter, Molly Gonzales, Glennon Mays

**Affiliations:** ^1^College of Veterinary Medicine and Biomedical Sciences, Texas A&M University, College Station, TX, United States; ^2^Department of Veterinary Integrative Biosciences, Texas A&M AgriLife Research, College Station, Texas, United States

**Keywords:** veterinarian-producer relationship, veterinary-producer perspectives, veterinary care barriers, education programs, partnership willingness, operation sustainability

## Abstract

**Objectives:**

To identify barriers to veterinarian-producer partnerships and suggest collaborative applied education as a means to enhance economic efficiency and sustainability of small and medium livestock operations and rural veterinary practices.

**Materials and methods:**

A participatory needs assessment, exploring the willingness and barriers to producer-veterinarian partnerships to enhance small/medium livestock operations, was distributed to Texas producers and veterinarians. Quantitative and qualitative data were collected via online, closed-ended survey questions and free response interviews. Responses were analyzed using SPSS and *HyperRESEARCH* to identify relevant terms, ideas, patterns, or themes.

**Results:**

Similar responses from 115 veterinarians and 58 producers revealed five major themes regarding relationship barriers: time, financial challenges, communication, competing perspectives, and respect. Overall producers reported greater willingness to partner in all areas, health care (90%), to achieve goals (80%), and to expand business (70%), than veterinarians. Veterinarian interviews revealed a need for increased animal health education among producers, while more than 60% of producers expressed high interest in continuing education on animal health topics.

**Discussion:**

Veterinarians and producers experience similar barriers to establishing partnerships. Both groups also recognize a need for education and prefer in-person collaborative learning communities Such educational opportunities can encourage formal veterinary-producer partnerships and provide solutions that enhance the economic efficiency and sustainability of small/medium livestock operations.

## 1 Introduction

Strong veterinary-client relationships are the hallmark of thriving veterinary practices. Producers of small and medium-sized livestock operations, especially in rural areas, prove a particularly challenging population of clients for veterinarians to build and maintain relationships with. Limited research exists describing the obstacles impeding relationship development. However, extrapolation from publications related to veterinarian and producer interactions indicates producers’ access to veterinary care, generational knowledge of producers, and the availability of veterinarians to provide service as prominent barriers. This research aims to clearly describe the challenges veterinarians and producers face in forming and sustaining partnerships and propose collaboratively led applied education programs as the means for creating, enhancing, and sustaining these relationships and the profitability of ranches and rural veterinary practices.

### 1.1 Access to veterinary care

Finances and negative perceptions limit access to veterinary care for producers of small and medium-sized livestock operations. Seeking veterinary care for individual animals is often determined by weighing perceived advantages against potential disadvantages (1). Does the animal’s market value outweigh the expense of a farm call? Could self-treatment provide a more economic recovery route? Producers must consider profitability when managing their operations. Though producers and veterinarians view animal welfare as a priority, managing animal health varies based on differing perspectives on economics and priorities (2). Producers hesitate to pay for veterinary care they deem unnecessary or cost-prohibitive, whereas veterinarians describe their time, knowledge, services, and products as prescriptive and reasonably priced (3).

Historically, producers demonstrate a reluctance to utilize resources provided by local veterinarians. They felt their opinions were unimportant, that veterinarians were uninterested in their operational goals and sought to profit from their needs. These perceptions are supported by findings from Degroot et al. (4) noting veterinarians rarely ask producers about their broader attitudes, ideas, goals, values, or motivations in making decisions. Moreover, veterinarians tend to communicate in a paternalistic style, taking on an expert role, and not treating the producer as an equal partner in the conversation. They relied on giving information and persuasion without making an effort to grasp the client’s perspective and experience (5). As a result, producers are disinclined to consult with veterinarians (6).

### 1.2 Generational knowledge

Multigenerational producers inherit not only the family farm but generations of knowledge regarding overall farm management, including care and treatment of livestock. Veterinary-producer relationships are hindered when producers use this generational knowledge to administer medications and vaccinations without consulting their veterinarian first (7). Veterinarians can assist producers in drug treatment options determined by an animal’s age, weight, breed, and underlying health conditions. Additionally, they provide knowledge of dosing schedules, drug interactions, and withdrawal periods. Producers lacking this guidance may provide incorrect dosage, administration, or off-label use which can adversely affect animal health and marketability.

Further, many food and drug products require veterinary authorization or administration. One example is the Veterinary Feed Directive (VFD) for agricultural use, passed by the Food and Drug Administration in 2015 (8). This was implemented to reduce the unnecessary use of medications in animals and to slow or prevent the development of bacterial resistance to antimicrobial drugs administered within medicated feed (8). Creating prescription-only requirements for medications and feeds has required producers to create and maintain veterinary relationships while allowing veterinarians to engage with producers to sustain their veterinary practices (7). Data-driven advancements in veterinary medicine are more reliable than generational knowledge. Current educational resources supplied through a strong veterinary-client relationship will benefit producer knowledge, and thereby operation profitability and sustainability.

### 1.3 Availability to provide service

Between 2010 and 2020 America’s rural population declined by 0.5%. Likewise, the number of farms decreased by 7% from 2017 to 2023 (9, 10). As a result, veterinary colleges face increasing difficulties retaining students with animal agriculture backgrounds most equipped to practice quality production medicine (11). Producers also prefer to partner with veterinarians who have strong farming backgrounds as they believe these individuals better understand the complexities of running a farm, as well as livestock medicine (12). Veterinarians must possess an in-depth knowledge of farm practices and business to earn the respect of producers (13). Additionally, many small and medium-sized farms support multiple species about which veterinarians may not be consistently knowledgeable. Hayes et al. (14) reported that a majority of veterinarians in their study population lacked confidence in treating multi-species due to insufficient exposure, experience, training, and/or knowledge.

Producers prefer to partner with veterinarians capable of making farm calls. However, veterinarians often find these visits impractical as they require specialized equipment and staff competent in handling livestock. Veterinarians lacking these resources are hindered in maintaining a rural practice (3).

In a study examining the sheep industry in the UK, researchers found that about two-thirds of ovine farmers only reach out to their veterinarian in the case of emergencies. They viewed veterinarians in the same regard as firefighters (15). Mindsets that include utilizing veterinary services only in emergencies, slow the development of trust and the creation of good partnerships (14), and thus the provision of services.

The current barriers between veterinarians and producers of small and medium-sized operations are ultimately the result of poor or non-existent relationships. To successfully develop these relationships there must be a renewed focus on understanding each other’s goals, challenges, and expectations. We propose that collaborative education opportunities can surmount these barriers and enable livestock producers and rural veterinarians to create strong relationships leading to sustainability and profitability for both parties.

## 2 Methods

This mixed-methods study focused on producers of small livestock operations and veterinarians practicing mixed or large production animal medicine in Texas. Two online surveys with closed and open-ended questions (Appendix A) aimed to explore the willingness of and barriers to producers and veterinarians creating partnerships to enhance the profitability and sustainability of practices/operations.

Texas A&M AgriLife Extension Services and Prairie View A&M University’s Cooperative Extension Program, an extension service whose mission is to respond to the needs of underserved Texans through learning opportunities that advance agriculture, promoted the producer’s survey. To achieve a representative sample, West Texas A&M University (WTAMU) Extension, Waller County Farmers’ & Ranchers’ Cooperative, and 100 Ranchers, Inc. assisted in recruiting producers of color with small livestock operations. The producer’s survey measured current and future interest in veterinarian partnerships, collected responses on the need for and awareness of local veterinarian services, and determined specific areas of educational need to maintain and sustain ranching operations.

Texas Veterinary Medical Association email listserv distributed the veterinarian survey. The veterinarian’s survey measured current and future interest in producer partnerships, collected responses on the awareness of producers’ needs and challenges, and determined specific areas of educational need to maintain and sustain local operations.

### 2.1 Data analysis

The closed-ended questions from veterinarian and producer surveys were analyzed using SPSS and the open-ended questions were analyzed using *HyperRESEARCH*. Open-ended responses were coded for keywords that aligned with question topics and identified areas of interest and research for this study. From the data reviews, a codebook was generated with clear definitions provided for each term or phrase. Codes that emerged during the data analysis were created to represent any term or idea that was deemed vital to the research (16). After reviewing the data, the researcher reflected upon the overall meaning of participant responses, identifying participant attitude and tone as well as patterns or themes. Results from the interpretation of the responses were represented using figures and tables and helped to inform further discussion of the findings.

## 3 Results

### 3.1 Quantitative survey responses

#### 3.1.1 Participant demographics

The study included 95 veterinarians: 52% female and 48% male, with a median age of 53. The racial composition was 96% Caucasian, 3% Hispanic, and 1% American Indian. These veterinarians have an average of 26 years of experience and operate small, large, mixed, and food animal practices across 68 Texas counties. In comparison, 58 producers responded to the survey: 60% male and 40% female, also with a median age of 53. These participants had more diverse racial backgrounds: 65% African American, 29% Caucasian, 4% Hispanic, and 2% American Indian. The producers manage operations with seven unique species with an average of 25 years of experience. It is important to note that some operations surveyed produce more than one species. Notably, the two study groups were almost identical in median age and years of experience but had greater variance in race and gender. Reliability of the quantitative questions of the veterinarian survey yielded Cronbach’s alpha = 0.85, while the producer survey yielded a Cronbach’s alpha = 0.80.

#### 3.1.2 Perspectives on service and information

Veterinarians significantly influence producers (17); however, not many veterinarians believe they do (14). Both groups of participants were asked about the services veterinarians offer and each group’s perception regarding how most producers seek out information about animal health.

Veterinarians reported providing 14 unique services to producers ranging from vaccinations and treatment to record keeping and financial management. However, producers primarily knew of only the four most common services—vaccinations, examinations, treatment, and urgent care. The percentage of producers aware of any services outside these ranged from 0 to 6.4%.

**Figure 1:**
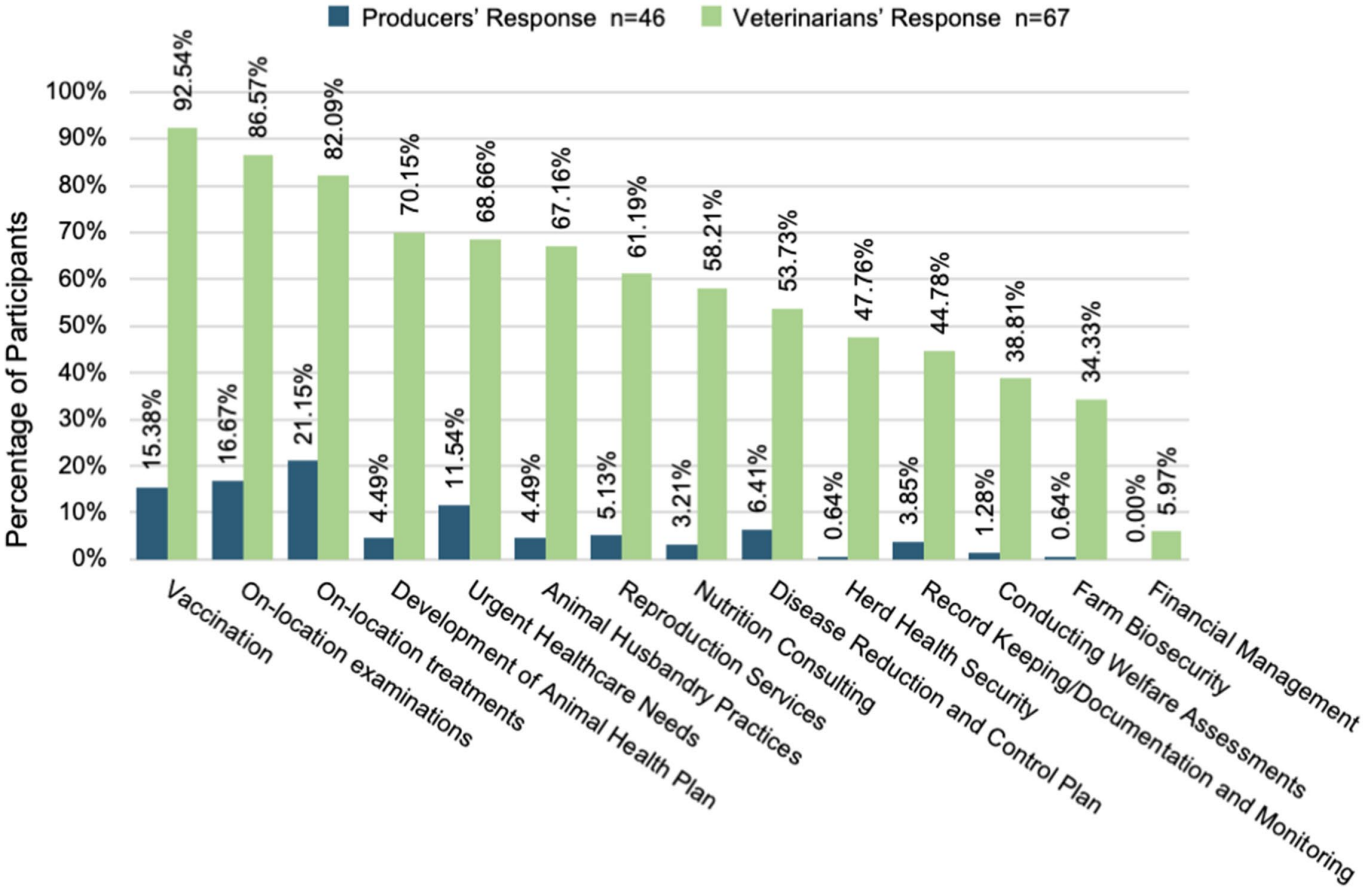
Perceived veterinary services offered.

**Figure 2:**
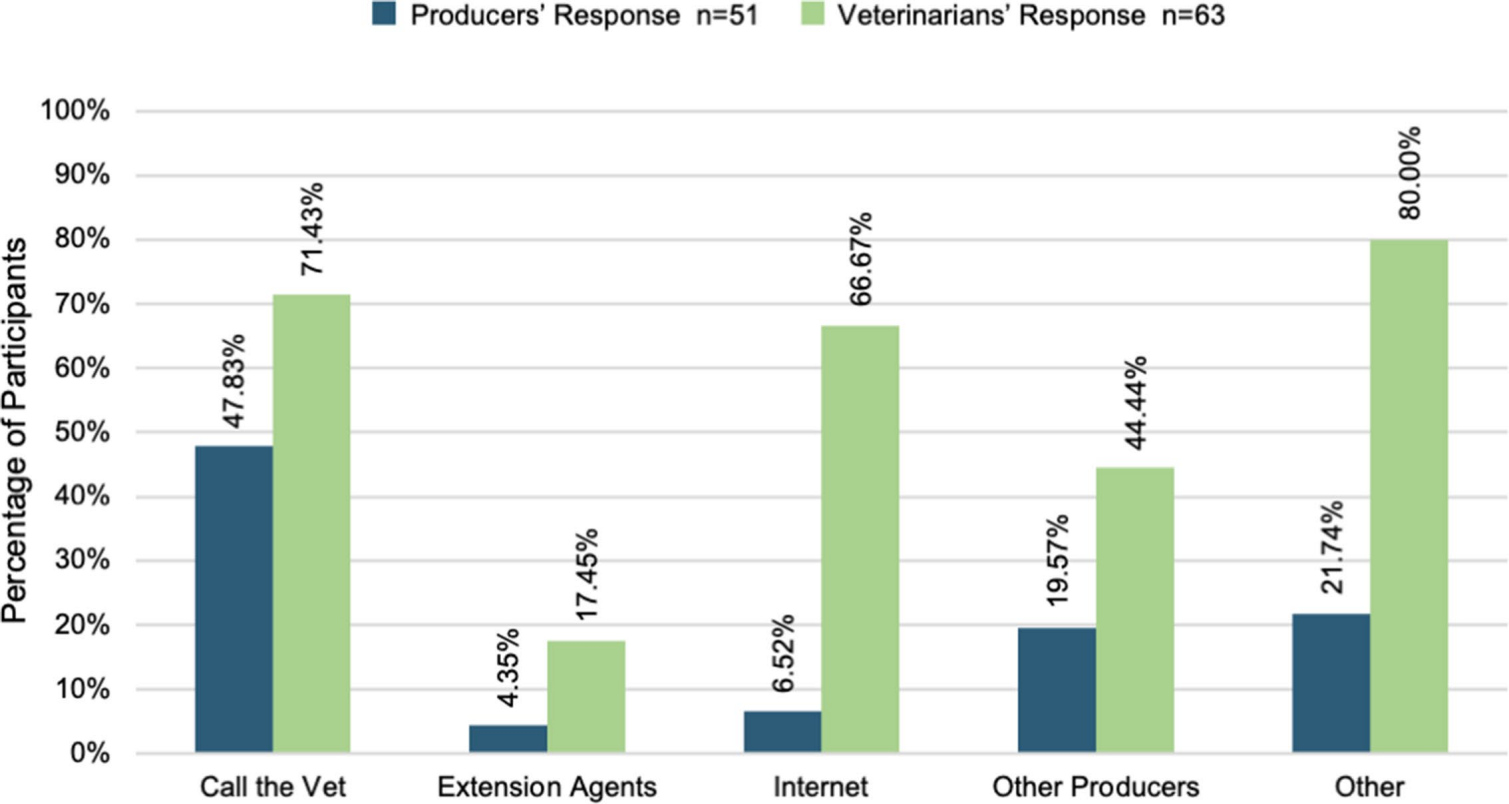
Animal health information sources.

Producers sought information from sources different from what the veterinarians perceived. Veterinarians expect only 24% of producers to seek their input about animal health questions when in reality twice that number, 48%, reported their veterinarian as their source of information for animal health. Veterinarians also expected producers to seek animal health information from the internet (22%) significantly more than the producers reported (6%). Figures 1 and 2 reveal the degree to which veterinarian and producer perspectives about available services and sources of animal health information are misaligned.

#### 3.1.3 Partnership willingness

**Figure 3:**
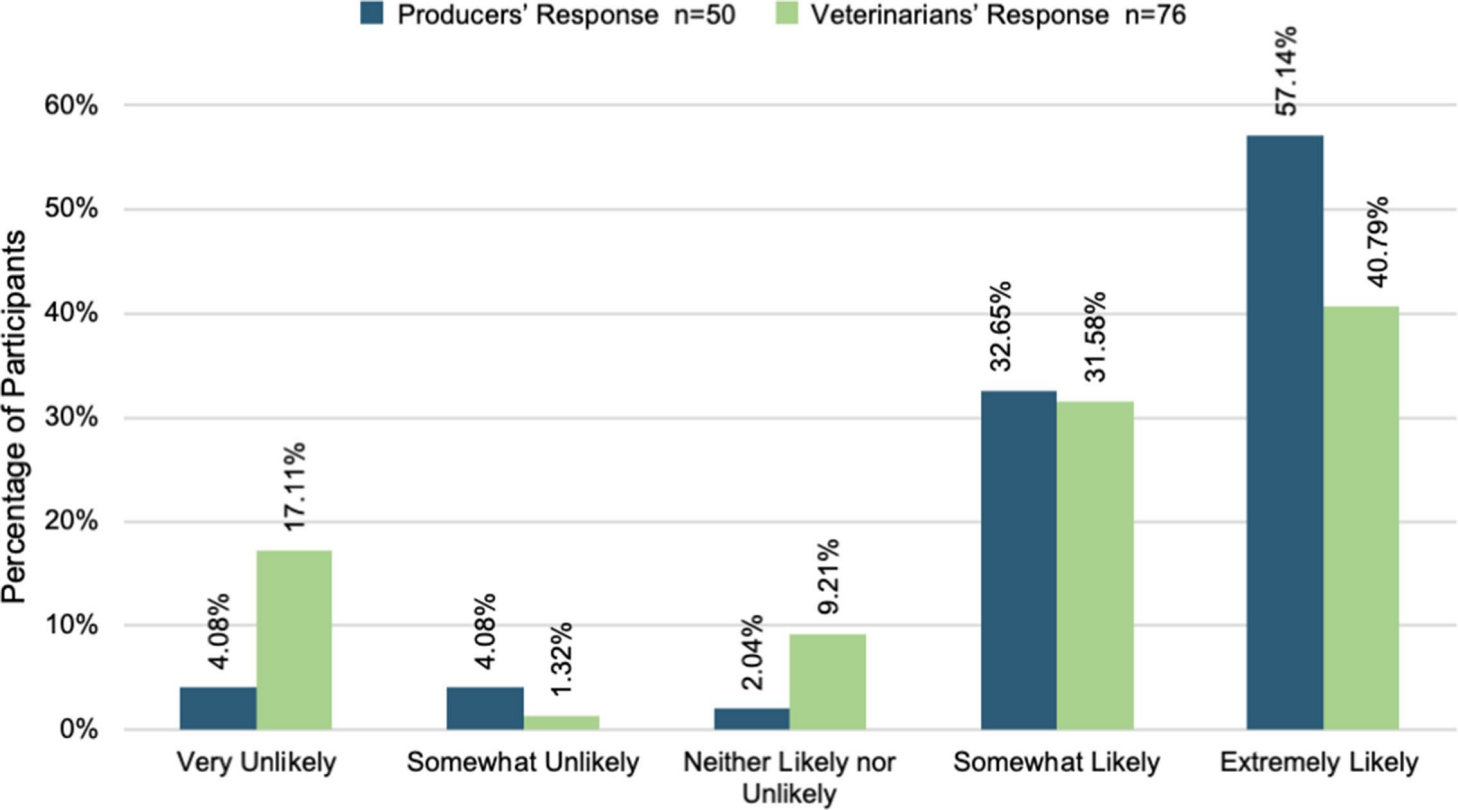
Willingness to partner for animal healthcare.

**Figure 4:**
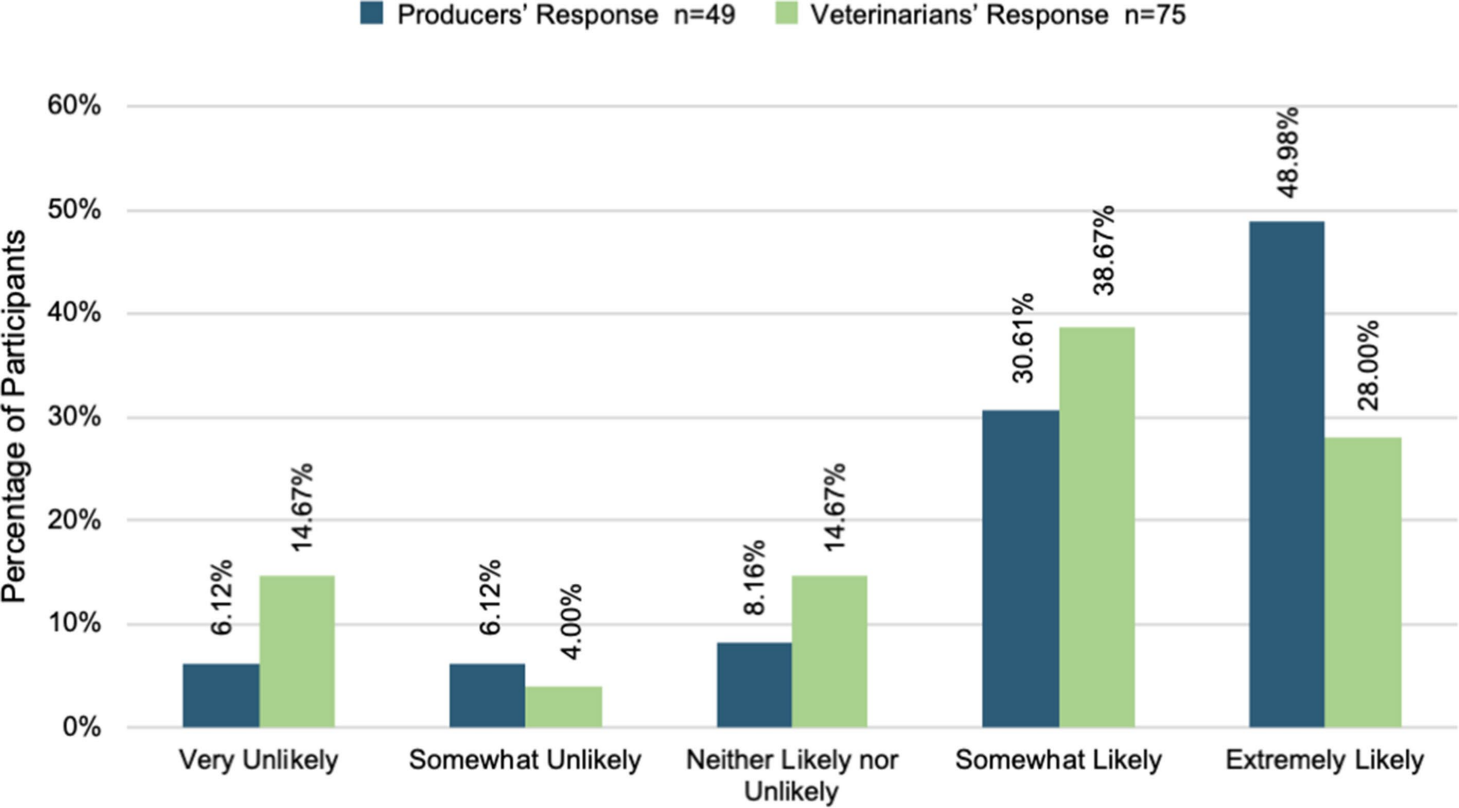
Willingness to partner to achieve goals.

**Figure 5:**
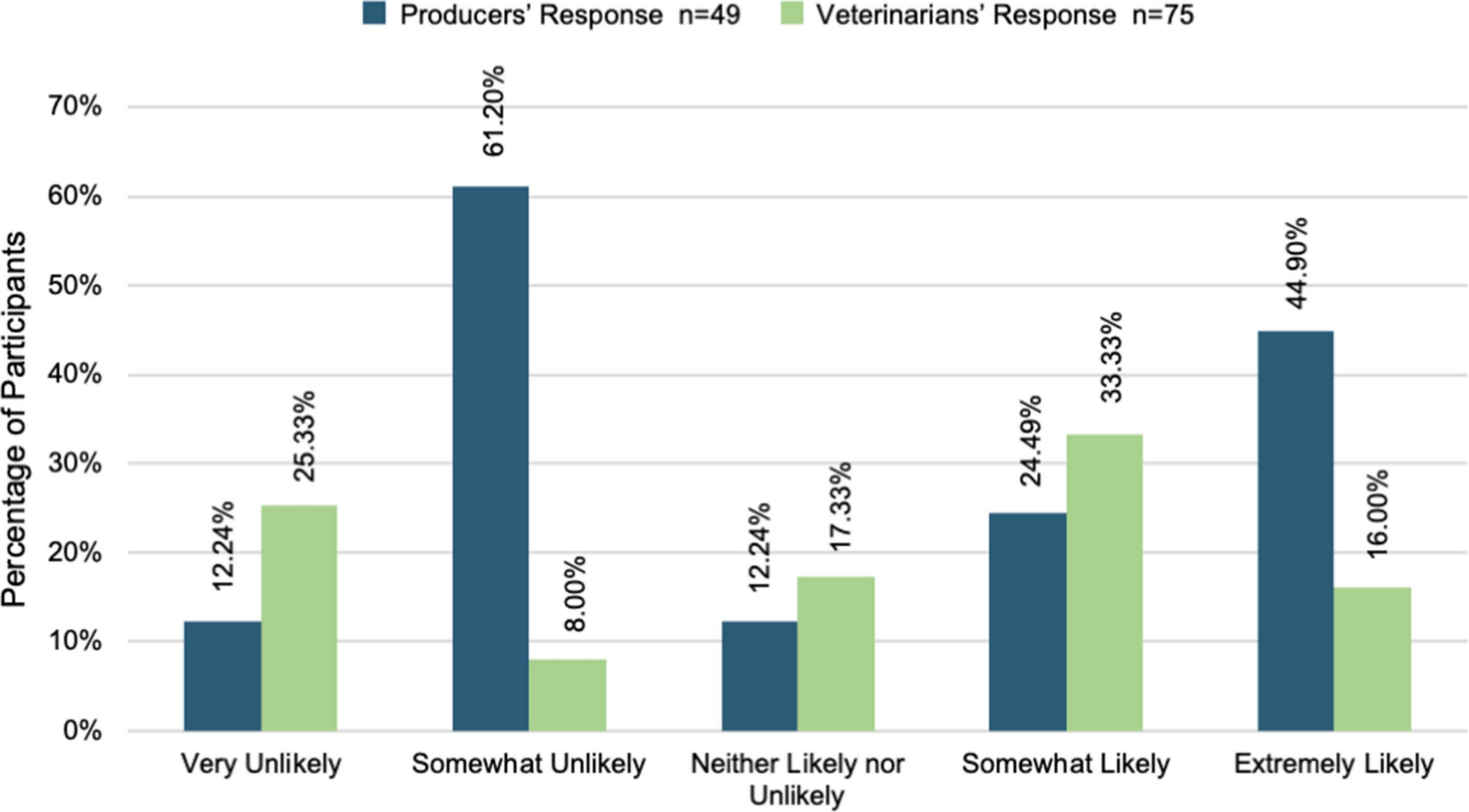
Willingness to partner to expand business.

Veterinarians and producers were surveyed independently on their willingness to partner with one another to provide animal healthcare, grow their businesses, and achieve their business goals. Most veterinarians and producers were willing to partner with one another to provide animal healthcare. Figure 3 shows that over 60% of veterinarians and producers were somewhat or extremely likely to partner for animal healthcare. However, a t-test comparing the two groups demonstrated a statistically significant difference between veterinarians and producers (t (123) = 2.43, *p* = 0.02, Cohen’s d = 0.43, CI [0.08, 0.80]). Producers (M = 4.34, SD = 0.87) were more willing to partner than veterinarians (M = 3.79, SD = 1.45) were willing to provide animal healthcare. Moreover, Figure 4 displays that most veterinarians (M = 3.61, SD = 1.34) and producers (M = 3.94, SD = 1.18) were willing to partner with one another to achieve their business goals. There was not a statistically significant difference found between the two group’s willingness in this area [t (121) = 1.40, *p* = 0.16]. Across all three areas, producers reported more willingness to partner with veterinarians in all areas. Furthermore, most veterinarians and producers were willing to partner with one another to grow their businesses (see Figure 5). On the other hand, a t-test comparing the two groups demonstrated a statistically significant difference in willingness veterinarians (t (121) = 2.22, *p* = 0.03, Cohen’s d = 0.41, CI [0.04, 0.77]). Producers (M = 3.67, SD = 1.39) were more willing to partner with veterinarians (M = 3.09, SD = 1.44) to grow their business than.

#### 3.1.4 Self-assessment of knowledge

**Figure 6:**
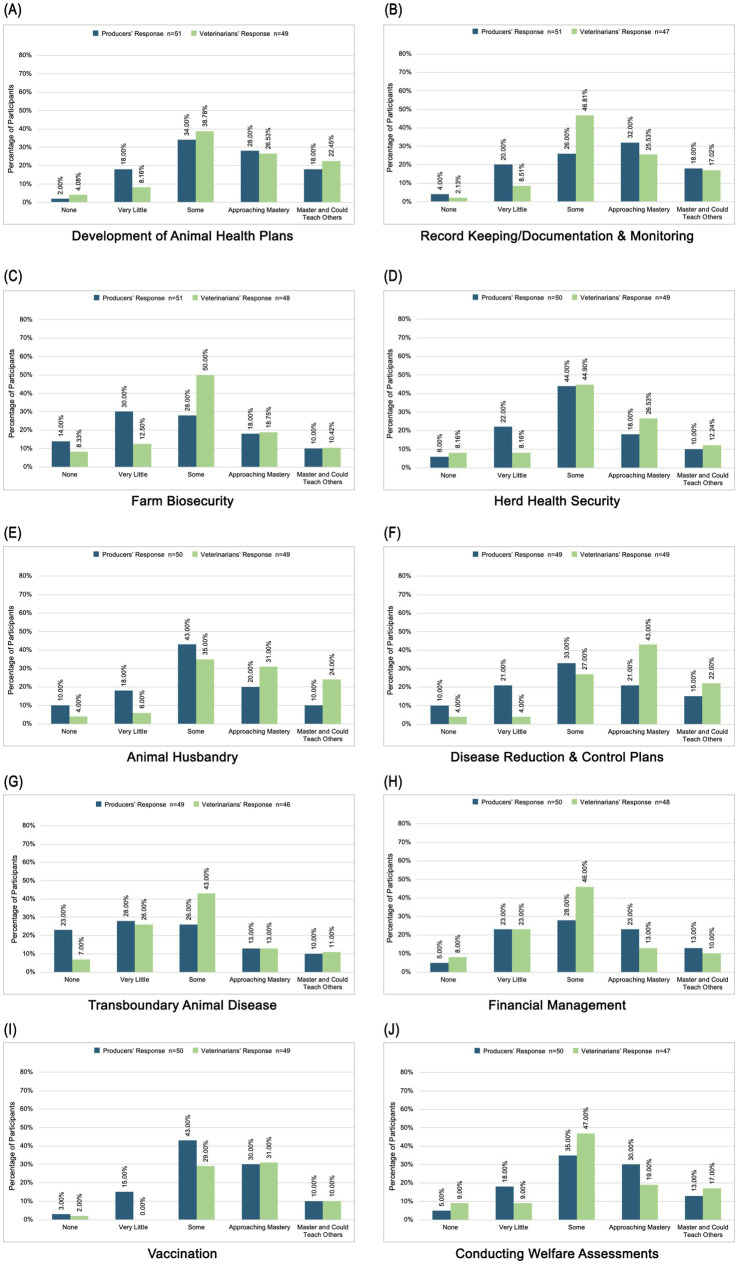
Self-assessment of knowledge in ten areas including **(a)** development of animal health plans, **(b)** record keeping/documentation and monitoring, **(c)** farm biosecurity, **(d)** herd health security, **(e)** animal husbandry, **(f)** disease reduction and control plans, **(g)** transboundary animal disease, **(h)** financial management, **(i)** vaccination, and **(j)** conducting welfare assessments.

**Figure 7:**
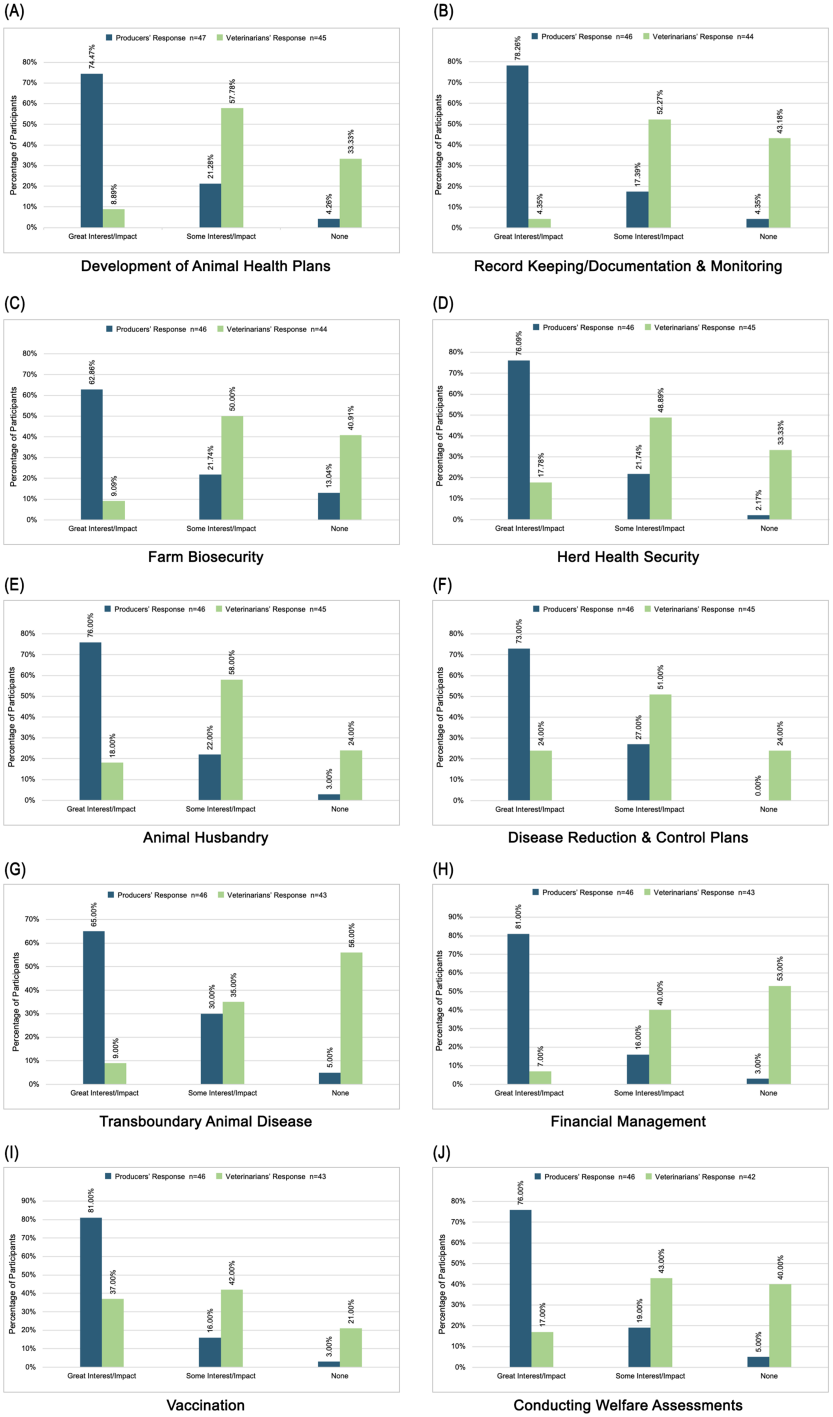
Impact of topic on developing a VCPR in ten areas including **(a)** development of animal health plans, **(b)** record keeping/documentation and monitoring, **(c)** farm biosecurity, **(d)** herd health security, **(e)** animal husbandry, (f) disease reduction and control plans, **(g)** transboundary animal disease, **(h)** financial management, **(i)** vaccination, and **(j)** conducting welfare assessments.

A literature review identified 10 animal health topics as possible avenues for veterinarians and producers to find common ground for developing partnerships. Veterinarians and producers indicated their current knowledge level on selected topics and producers indicated their interest in educational resources for these topics while veterinarians rated the impact such resources might have on the veterinary-producer relationship. Less than 40% of producers assessed their level of knowledge as “approaching mastery” or “master” for seven out of ten topics (“approaching” 13% ≥ x ≤ 32% and “master” 10% ≥ x ≤ 18%). However, a majority (63–81%) expressed a high level of interest in pursuing continuing education on all topics. Greater than 40% of veterinarians assessed their level of knowledge as “approaching mastery” or “master” for six out of ten topics (“approaching” 13% ≥ x ≤ 43% and “master” 10% ≥ x ≤ 39%). Most of their responses (46–79%) signified that continuing education in all topics would positively impact the veterinarian-producer relationship. Figures 6 and 7 describe these topics and participant responses.

#### 3.1.5 Professional development training preferences

**Figure 8:**
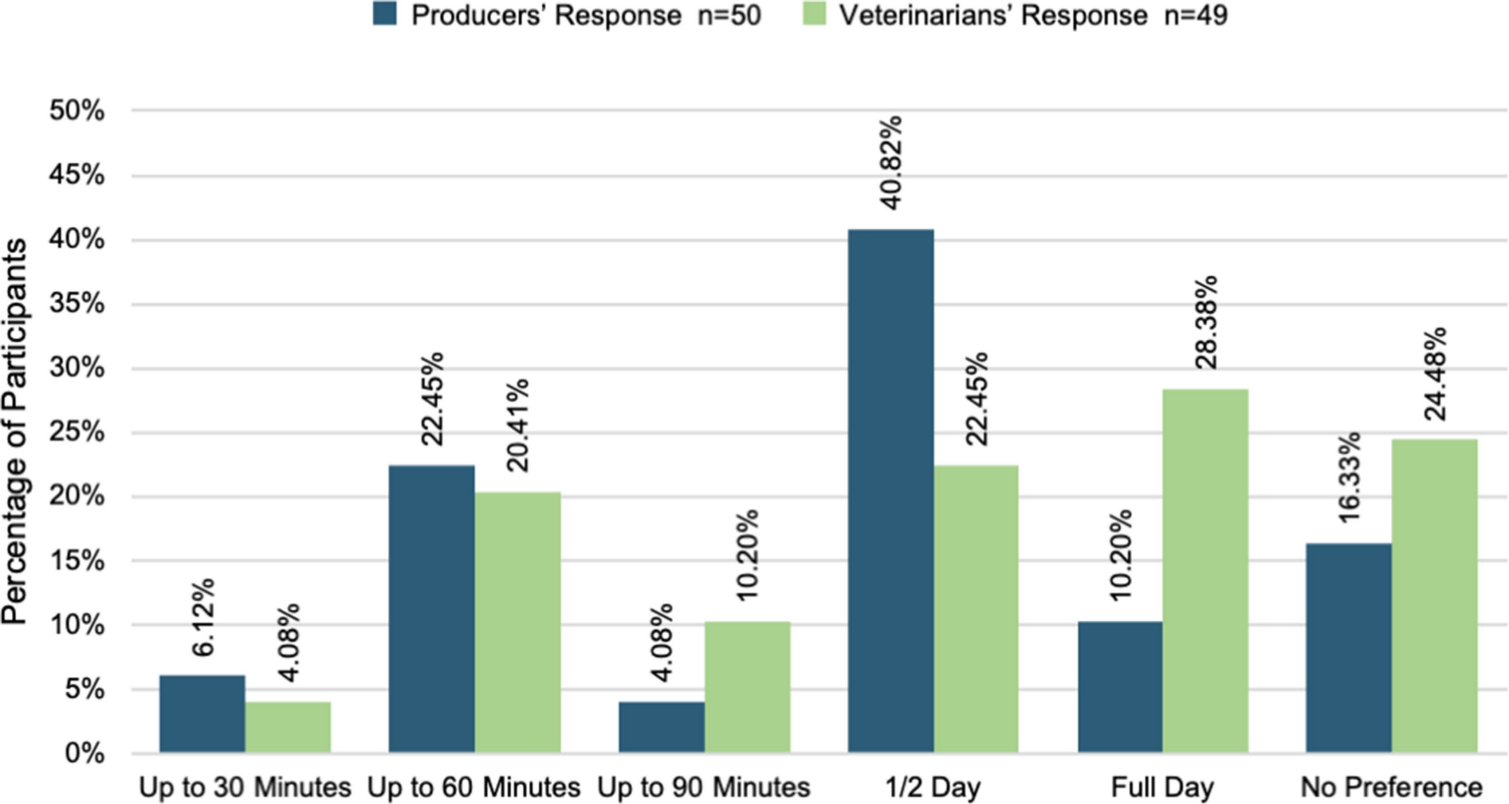
Preferred length of time for in-person training.

Veterinarians and producers also ranked preferences for continued education training and learning styles including length of time for in-person training. Both groups ranked “in-person learning communities” as most effective with a half-day time frame most preferred (22 and 41% respectively, Figure 8). Tables 1 and 2 describe training preferences.

**Table 1:** Learning environment preferred by veterinarians.

Learning environment	Least effective	#	Somewhat effective	#	Most effective	#
Participating in an in-person learning community (e.g., monthly, or quarterly)	2.68%	3	9.22%	26	20.20%	20
Presentation(s) followed by discussion	1.79%	2	10.28%	29	18.18%	18
Workshops to address challenges	6.25%	7	9.57%	27	15.15%	15
Workshops to apply learning/complete an activity at session	5.36%	6	10.64%	30	13.13%	13
Online self-paced modules	15.18%	17	7.80%	22	10.10%	10
Informal discussions on designated topics	7.14%	8	11.35%	32	9.09%	9
Workshops to work on projects (e.g., group or individual)	11.61%	13	10.28%	29	6.06%	6
Online facilitated modules	14.29%	16	10.28%	29	4.04%	4
Online sessions using collaborative meeting software	19.64%	22	8.87%	25	2.02%	2
Participating in an online learning community (e.g., monthly, or quarterly)	16.07%	18	10.64%	30	1.01%	1

**Table 2:** Learning environment preferred by producers.

Learning Environment	Least effective	#	Somewhat effective	#	Most effective	#
Participating in an in-person learning community (e.g., monthly, or quarterly)	7.32%	3	24.39%	10	68.29%	28
Presentation(s) followed by discussion	16.67%	8	29.17%	14	54.17%	26
Workshops to address challenges	18.37%	9	32.65%	16	48.98%	24
Workshops to apply learning/complete an activity at session	2.50%	1	50%	20	47.50%	19
Online self-paced modules	16.67%	8	39.58%	19	43.75%	21
Informal discussions on designated topics	27.50%	11	37.50%	15	35%	14
Workshops to work on projects (e.g., group or individual)	15%	6	52.50%	21	32.50%	13
Online facilitated modules	25%	10	42.50%	17	32.50%	13
Online sessions using collaborative meeting software	15.79%	6	52.63%	20	31.58%	12
Participating in an online learning community (e.g., monthly, or quarterly)	33.33%	13	35.90%	14	30.77%	12

### 3.2 Interview results

Upon survey completion, participants were invited to participate in a follow-up interview (Appendix B) conducted by a research team member. The responses were collected for analysis alongside the initial survey responses. Producers elaborated on barriers to sustaining operations, perspectives on existing relationships with veterinarians, and interest in pursuing and establishing a partnership with a veterinarian. Veterinarians who maintain a practice of less than 90% small animals were invited to participate in the follow-up interview. These veterinarians discussed the challenges faced in sustaining their practice, described relationships with producers, and offered perspectives on creating producer partnerships. Veterinarian and producer responses were analyzed separately for each type of data. The two sets of findings were compared to explore similarities and differences in perspectives and experiences. Both quantitative and qualitative findings for each group were integrated to arrive at study conclusions.

#### 3.2.1 Veterinarian interviews qualitative findings

Fifteen veterinarians responded to 12 open-ended interview questions whereby they addressed thoughts regarding their practice, the type of care provided, perspectives on client-veterinarian relationships, and willingness to partner with producers. The twelve interview items produced a total of 531 coded passages which were further categorized into the following 10 themes. Within each theme, subthemes were identified (Appendix C). These themes, accompanied by respondent quotes, are presented below.

##### 3.2.1.1 Theme 1: practice description

Respondents were asked to describe their current practice, including species seen. The responses revealed 12 subthemes regarding veterinarians’ practice. These 12 subthemes were utilized 102 times when coding participant responses. Eleven out of fifteen veterinarians cited having a mixed animal practice, and only four practices were exclusively large animals. While all 15 veterinarians noted working with equine clients, over half of the participants also noted working with other species including bovine, swine, and small ruminants.

##### 3.2.1.2 Theme 2: practice sustainability challenges

Veterinarians were asked about the challenges faced in maintaining and sustaining their practice. The theme of “Practice Sustainability Challenges,” resulted in 55 coded passages with seven subthemes. Across the responses, veterinarians spoke to the growing challenges of sustaining their practices due to the cost of care, practice maintenance, and limitations of facilities lending to limited-service offerings. In 12 instances, interviewees spoke about the economics of veterinary medicine and the challenge of limited funds and resources. One participant stated, “Everyone wants to take the animals to the vet, but the cost–benefit is not there for food species…the horses and cats, it is still there but it is still an economic issue.”

Many veterinarians noted that while they would prefer to be a solely large animal practice, however, veterinary medicine is not subsidized, and “the economics of what it costs (the practice) versus the value of the animal is often not compatible with producers seeking veterinary care.” Thus, many veterinarians feel forced to turn to mixed animal practices to help make ends meet. “If I were strictly doing production medicine, then that would be wonderful. But no one can do that and make a living. So, I have to open my practice to dog and cat health now and that takes up time.”

##### 3.2.1.3 Theme 3: barriers to providing veterinary care

Veterinarians were asked questions related to how producers’ provision of animal care has impacted their practice, as well as how relationships with producers influence their response to after-hours calls. Fifty-five coded passages highlighted the theme of barriers to providing veterinary care, and seven sub themes emerged. Of the 55 coded passages, statements relating to limited profit and time were the most abundant. For veterinarians, dependent upon an operation’s size and care needed, they could see minimal financial gain in providing services. Additionally, many veterinarians held the perception that limited profit for the producer also presents a barrier to providing veterinary care. “In the producer’s mindset, it is always a cost. This leads to resistance. There’s an economic value in the animal that if your procedure exceeds that breakeven point, it is economically unproductive to do the procedure.”

In addition to limited financial gain, time was also seen as a limiting factor. Five of the 15 respondents noted that they have placed boundaries around their time and mental health in an attempt to achieve work-life balance. For many, expectations to work after hours or on weekends is a strain on their family and so they have chosen to limit their availability. According to one veterinarian, “I will not provide services outside of my hours. We have enough demands on our time, our family, our mental and physical health.”

Additionally, many veterinarians noted that ‘not enough time’ is a major challenge. Due to the high demand and limited availability of veterinarians, many producers have chosen to provide care themselves.

##### 3.2.1.4 Theme 4: incentives to seeking veterinary care

Veterinarians were asked about incentives available for producers to engage with their veterinarian rather than handling herd health independently. The theme of Incentives to Seeking Veterinary Care resulted in 24 coded passages with four subthemes. Over half of the veterinarians interviewed spoke to the importance of producers having a veterinarian on record to request prescriptions. One stated, “Veterinary feed directive and prescription medications [are incentives]. We are obligated to have client and patient relationships, and some vets, sadly, do not follow that.” Another shared, “You have to be careful as a vet and aware that people will call and want to get medicine and script for feed additive. If there is no relationship with a client and doctor, then I will have to say no to them.” Several also noted that being on record often results in a smoother process for accessing needed care.

##### 3.2.1.5 Theme 5: federal programs

Veterinarians were questioned about veterinary oversight in the form of federal programs like the Veterinary Feed Directive. Participants were asked how federal programs have influenced veterinary oversight in their practice. This line of inquiry served as the foundation for 28 coded passages, and seven subthemes. Eleven out of fifteen veterinarians stated that federal programs have not impacted their practice, while three noted a positive increase and one a negative impact. When asked how federal programs influenced their practice, one veterinarian noted, “No change. I do not know of anyone that is concerned with feeding medicated feed. I have not seen it. Barely been asked about it.” Another veterinarian felt federal programs had a positive association, stating, “I think that the change in the veterinary feed directives is a very good thing. I understand that it can change some of the producer outcome[s], but I am adamantly in line with the antimicrobials. We have got to protect our antibiotics. This has not affected my practice.” This same veterinarian was quick to note that federal programs have streamlined how producers obtain medicated feed and medicines. However, other veterinarians were open about the challenges that remain in enforcing these programs. “The Veterinary Feed Directive has not changed our relationship much. Those who used the medications before are still coming to us for the feed. Those that did not come to us in the past still do not come. We’re close to the border so there are lots of unlicensed practices along the border, and the state does not have the teeth to stop it. Those that do not want to have the relationships with the vet can still skirt the system.”

##### 3.2.1.6 Theme 6: producer provided animal care

Veterinarians were asked how producer-provided livestock healthcare has impacted their practice, animal well-being, and animal health. These questions resulted in 50 codes across eight subthemes. When questioned about how producer-provided livestock health care has affected their practice, nine out of fifteen shared that this had, “Economically, no effect at all. It does not affect me.” However, several expressed some frustration in noting that producers have varied knowledge and skill sets when it comes to taking care of their animals. This results in producers contacting veterinarians only in emergencies. One veterinarian noted, “Smaller mom-and-pop producers seem to only reach out to veterinarians during emergencies.”

Regarding the lack of veterinary oversight impacting animal well-being, over half of respondents felt that there was no impact, with one respondent expressing a negative impact. Similar to their feelings regarding the effect of producer-driven care on their practice, a majority of veterinarians expressed their main concern being producers’ varied levels of knowledge and skills. “I think a lot of it is lack of education even in some very educated people…Some of the animal welfare issues we see are not from an intentional standpoint but there is just a lack of education…I do not have a problem with people doing some things, but at the same time there needs to be that level of education and that level of cooperation between a producer and veterinarian.”

##### 3.2.1.7 Theme 7: strategies to mitigate impact of producer-provided animal care on practice

Interviewees were asked to describe strategies that they have used to mitigate loss associated with producers providing their own animal care. Responses to this question led to 48 coded passages and four subthemes. Many veterinarians in early questioning expressed that little impact was felt by their practice; although, several shared strategies that they feel could result in a positive impact. Twenty coded passages focused on the value and importance of veterinarians providing timely responses and quality service. With time being a recognized barrier to the provision and seeking of care, many noted the importance of finding a better balance with their time to meet the needs of their clients. One veterinarian noted, “Give them better service. Answer the phone when clients call. The biggest complaint is producers cannot get the vets to call them back or come to visit their farm/ranch in a reasonable time period.”

Participants also noted the importance of communication as the foundation of any relationship ultimately becoming the catalyst for developing new and improving existing relationships. Finally, 17 coded passages noted the importance of providing client education. “I think there needs to be a fair amount of education pushed. We need to have one-on-one conversations with these producers to let them know what we can provide different from others.” Another veterinarian stated, “I try to be more informative with my clients and educate them on best care practices and why we need veterinarians instead of asking the internet for help.” In addition to educating clients on the importance of veterinarians, some also noted that educating clients on procedures they can perform safely on their own, could promote partnerships with producers and set them up for success.

##### 3.2.1.8 Theme 8: partnerships with producers

Veterinarians were asked if they would be willing to partner with producers to enhance their veterinary practice. Additionally, they were asked to describe what a partnership with a producer would look like. These two questions led to 48 coded passages and the development of seven subthemes, though one of the subthemes “no relationship—potential for delayed care,” was utilized for responses to other interview items. All veterinarians interviewed shared their willingness to partner with producers. When asked what a partnership would look like, 10 participants shared that consistent communication would be key in establishing knowledge of a client’s operation and needs. Additionally, six veterinarians expressed a desire to offer programming and education as part of the relationship, to ensure that they were supporting their clients in understanding best practices for care and overall herd health. For many veterinarians, partnerships form a community where clients are not just customers but friends as well. One veterinarian noted, “I live in a town with 1,300 people and about 5,000 in the county. The same people who are your clients are the people you hang out with, socialize with, and go to church with. There is a bond there.”

While all veterinarians recognize that each producer partnership would be unique, they all expressed a desire to establish relationships built on mutual respect and a better understanding of one another.

In expanding on the topic of producer relationships, veterinarians were asked how established producer relationships impact your response to care outside of scheduled appointments or after-hours calls. Almost all veterinarians agreed (12 out of 15) that they would hesitate or choose not to see a client after hours unless there was an established relationship. While many want to help, they also want to maintain personal boundaries, especially when they have limited knowledge of a client’s needs. One veterinarian stated, “I am much more able to help someone I have a working relationship with rather than an emergency relationship.”

When describing relationships with producers outside of scheduled care, veterinarians reported various levels of relationships, with many noting that they are part of the same community as their clients. This leads to interactions outside of scheduled care that are most often noted as amiable or friendly. A respondent summarized the spectrum of responses when they stated, “It’s no different than with people that are accountants or lawyers or teachers. Some are sociable friends, some are acquaintances, and some you never see outside your business.”

##### 3.2.1.9 Theme 9: methods for fostering/maintaining relationships with producers

Veterinarians were asked how they create and maintain producer relationships. Responses to these interview items resulted in 71 coded passages highlighting the theme. Through this theme, five subthemes emerged. Twelve out of fifteen veterinarians interviewed felt that building and maintaining trust is key to cultivating relationships with producers. One interviewee stated, “You have to partner with them, and you want your owners and producers to be as successful as they can be within their own limitations. I want to be on the asset side of the ledger, not the liability.” Other respondents spoke to the importance of being authentic, treating producers with respect, and showing them how you can be of value to their operation. Nine veterinarians discussed the importance of increased collaboration, where ‘it looks like a family relationship with two-sided equal and mutual respect.’ When veterinarians and producers respect each other’s roles within the relationship, it creates an environment conducive to collaboration and establishing shared goals for the operation.

In addition to trust and collaboration, veterinarians expressed that hosting seminars and training related to animal health and production could serve as gateways to establishing and maintaining producer relationships. By offering educational opportunities to grow knowledge and skills, veterinarians can help educate producers in areas that are relevant to their operations.

##### 3.2.1.10 Theme 10: limitations to relationship development with producers

Veterinarians were also asked what might limit them from developing producer relationships. This question resulted in 44 coded passages and the emergence of seven subthemes. Every veterinarian expressed at least one limitation related to the lack of time for relationship development. Many recognize how limited their time is already, not including the additional efforts needed to establish and develop new relationships with producers. One veterinarian stated, “there is not enough time to get things done” while another shared that things could be different if they, “had more time, which would then allow for more availability.” In addition to time, veterinarians also alluded to financial burdens and profit limitations that can come with creating producer relationships. One veterinarian noted sadly that, “It all revolves around money at the end of the day.” While all veterinarians want to help, it is also understood that the size of the operation and the type of care being requested influence whether that work is profitable. Another participant noted, “Not sure about food animals, but the value of that animal has not kept up with the need to charge what we need to charge in order to make a living with the service…I have to charge what my time and energies are worth these days to make it worth it and not get taken out of the market.”

The next section addresses the producer population and their interview responses.

#### 3.2.2 Producer interviews qualitative findings

Twenty-two producers responded to 12 open-ended interview questions wherein they expressed views related to their operation, species of animals cared for, perspectives on veterinarian relationships, and willingness to partner with veterinarians. The 12 interview items produced a total of 407 coded passages which were further categorized into the following nine themes. Within each theme, subthemes were identified (Appendix D). These themes, accompanied by respondent quotes, are presented below.

##### 3.2.2.1 Theme 1: operation description

Producers were asked to describe their current operation, including what species they raise. Through the responses provided, 10 subthemes emerged. These 10 subthemes were utilized 52 times when coding participant responses. Eight out of twenty-two producers cited having a small operation, with all but three producers raising bovine as part of their operation. In addition to raising bovine, participants also noted raising equine, swine, poultry, and small ruminants, with equine being the second most popular. A few producers also noted that they are Next Generation producers eager to carry on their family legacy.

##### 3.2.2.2 Theme 2: operation sustainability challenges

Producers were asked about the challenges they face to maintain and sustain their operations. The theme of “Operation Sustainability Challenges,” resulted in 62 coded passages with five subthemes. Across all responses, producers spoke to the growing challenge of sustaining their operations and herds due to the exponential increases in the cost of care and overall cost of the operation. In thirty-one instances, interviewees spoke to rising overhead costs for their operations and the resources needed to care for their animals. With limited funds and resources, many producers are finding it challenging to acquire and maintain the equipment and facilities needed, while also providing appropriate animal nutrition. One participant stated, “Challenges are high with infrastructure cost and equipment, high fertilizer cost, and wild hog damage to the pasture.”

In addition to the cost of care, one producer noted that it was challenging for them to find a veterinarian to work with their swine, “There is not a veterinarian in the area that specializes in pigs. Everybody kind of does it because they have to. It’s hard to find someone who knows about pigs and is willing to actually work with them.”

Another producer spoke about lacking reliable transportation to get animal care should they need it. While noted less, some producers also mentioned difficulties due to pests, like wild hogs, and harsh weather. These producers spoke at length about the challenges they are facing with the current drought and having to decide to reduce herd size due to a lack of hay.

##### 3.2.2.3 Theme 3: barriers to seeking veterinary care

Producers were asked why they choose to provide their own care instead of consulting their veterinarian, as well as how their relationship with a veterinarian influences responses to calls for after-hours care. Fifty-eight coded passages highlighted this theme, and 10 subthemes emerged. Of the coded passages depicting why producers often choose to provide their own care, statements relating to financial burden and preference for providing one’s own animal care were the most abundant. For eighteen out of twenty-two producers, the financial burden brought on by the cost of veterinary care lends to the overwhelming preference to provide their own animal care when possible. One producer stated, “I do have a veterinarian provide the service sometimes, but I provide this care because of financial reasons. They [cattle] cost $50–$100 per head, and by the time you pay the chute fee and vaccination cost, it’s at least $150 per head per cattle at that point.”

Other producers noted time as a significant barrier. Producers often find it challenging to connect with veterinarians, or the time and distance associated with transporting the animal to receive care is not sustainable. “It’s easier when it comes to scheduling, and it’s cheaper. I like to do things by myself. I’m already home.”

In this absence of a standing veterinary relationship, producers were asked to describe the impact on after-hours/emergency response. Two of the interviewees spoke about the potential for delayed care if they are not on file with a veterinarian. “If you wait until the middle of an emergency, it’s too late to find somebody to help you…If you have to develop that relationship during an emergency, more than likely you will not survive the emergency.” Another shared that in times of an emergency, they will “usually try to call a vet, and if no response I’ll handle [it] on my own.”

##### 3.2.2.4 Theme 4: incentives to seeking veterinary care

Producers were asked about the benefits provided by veterinary involvement, as well as incentives available for engaging with their veterinarian. The theme of Incentives to Seeking Veterinary Care resulted in 44 coded passages with five subthemes. Over half of the producers (*n* = 15) interviewed shared that they were not aware of any incentives available for them to engage with their veterinarians. All 15 simply responded, ‘No.’ When asked about the benefits of veterinary involvement, 11 expressed the advantage of having veterinarians consult on their operation and overall herd health. One stated, “Primarily consulting. Like for illnesses, especially on the pigs. If one is showing signs of an illness, then being able to call and text a veterinarian for guidance is very beneficial.”

Another shared, “You can call them, and sometimes, for instance, I had a cow have a fungus on his head, and I took a picture and sent it to the vet so we could talk about it over the phone.” Several also noted that veterinary involvement provides them with access to more knowledge, on-site care, and assistance in securing prescription feeds or medications when a veterinarian is on record.

##### 3.2.2.5 Theme 5: federal programs

Producers were questioned about the regulation of veterinary oversight in the form of federal programs like the Veterinary Feed Directive (VFD). Participants were asked how federal programs have impacted their livestock operations. This line of inquiry served as the foundation for 15 coded passages and one subtheme. Fifteen out of twenty-two producers stated that federal programs have not impacted their operations. They have an established record with the vet to obtain medicated feed or prescriptions when needed. The seven remaining producers chose to not answer this question. One nutritionist/producer noted that many veterinarians are not truly knowledgeable of programs like the Veterinary Feed Directive, creating conflict when questions arise. “As a nutritionist though, there have been some challenges. The biggest challenge is veterinarians do not understand the feed law. They are not ‘educated’ in the proper way of writing a veterinary feed directive. When you have an issue, veterinarians are very busy and it’s hard to get them to focus on questions and issues you have with a VFD.”

##### 3.2.2.6 Theme 6: producer provided animal care

Producers were asked about their experience with providing their own animal care including what type of care they provide. Twenty-two coded passages highlighted this theme, and four subthemes emerged. All twenty-two producers shared that they provide some form of their own animal care, whether pest management (i.e., deworming), production management (i.e., castration, etc.) or vaccinations. One producer shared that providing this form of care was in their blood, as these skills have been passed down from previous generations and a veterinarian is not needed. When asked to describe their experience with providing their own care, 14 producers noted that providing their own animal care is easy and sustainable. One producer stated, “Most of the time, it’s pretty easy” while another stated, “Self-service is the best.” Meanwhile, five producers did note that providing care has its challenging moments and they will turn to their veterinarian in times of emergency. A participant confessed, “It can be challenging and tough doing it yourself.”

##### 3.2.2.7 Theme 7: partnerships with veterinarians

Producers were asked to describe the veterinary relationship outside of scheduled care; willingness to partner with a veterinarian to enhance your livestock operation; and what a veterinary partnership looks like. These three questions led to 57 coded passages and the development of nine subthemes. All producers interviewed minus one stated a willingness to partner with veterinarians to advance their livestock operation. When asked what a partnership would look like, 16 participants commented that consistent communication would be key to developing a relationship with a veterinarian such that they could become knowledgeable of their operation and overall herd. Many producers recognize that a veterinary relationship can give them greater insight into their herds’ needs. For many producers, they also stress the importance of creating a relationship built upon mutual respect. One producer noted, “I think that it’s important for the veterinarian to be priced fairly and for the producer to pay his bill promptly and not expect anything for free. There should be mutual respect between the two. Respecting [the] time of both people.”

Like the quote above, six producers expressed wanting to have a partnership where the veterinarians provide necessary care and they in turn value the cost of that veterinarian’s time and services. When asked about their relationship with their veterinarian outside of general scheduled care, three producers noted having no relationship, and 13 producers had a limited relationship outside of care. Four producers expanded further stating that the veterinarians are a part of their community. One producer noted, “It’s a really good relationship because we get to communicate and get to see them at almost all gatherings.”

##### 3.2.2.8 Theme 8: methods for fostering/maintaining relationships with veterinarians

Responses to interview questions related to creating and maintaining veterinarian relationships resulted in 27 coded passages highlighting the theme. Through this theme, five subthemes emerged. To foster relationships, six producers pointed to consistent scheduled care as a means to establish a working relationship with the veterinarian. From there, three noted it is up to both veterinarians and producers to keep communication flowing. Twelve producers discussed the importance of increased collaboration, where the partnership results in ‘trusting the person who will help take care of my animals.’ In addition to collaboration and communication, producers expressed the value of educational opportunities to improve their knowledge and skills. One producer stated, “It would be nice to be available and have semi-annual or annual meetings between veterinarians and producers.” These gatherings would promote education about regional animal health issues and aid producers in knowing when veterinarian involvement may be necessary.

##### 3.2.2.9 Theme 9: limitations to relationship development with veterinarians

Producers were also asked to describe the limitations of developing veterinary relationships. This question resulted in 26 coded passages and the emergence of one subtheme. Every producer noted “time” as the primary limitation to nurturing a relationship with their veterinarian, whether their time constraints or the perceived time constraints of their veterinarian. One producer stated, “The hardest thing is just how busy veterinarians are. There is a huge demand for large animal veterinarians, and the more knowledgeable they are, the more busy they are. Being able to get that time, especially when dealing with pigs, he [the veterinarian] tries to fit him in when he can between horse clients. That is a challenge. Regardless of what they do, every veterinarian I’ve talked to is just busy with helping their other clients.” Another producer reflected on their capacity for relationship development and noted that while they are interested in advancing their operation, they are, “Not available to do so.”

## 4 Discussion of key findings

Data analysis resulted in multiple findings related to veterinarians’ and producers’ perspectives on the value of relationships and the overall impact on animal health. The analysis of quantitative findings supports the analysis of qualitative findings for both populations. In addition, the responses provided by both veterinarians and producers were similar. The coded passages across both populations were grouped into the following 10 main themes: (a) practice/operation description, (b) practice/operation sustainability challenges, (c) barriers to seeking/providing veterinary care, (d) incentives to seeking veterinary care, (e) federal programs, (f) producer provided animal care, (g) strategies to mitigate impact of producer provided animal care on operation (unique to veterinarians), (h) partnerships with veterinarians/producers, (i) methods for fostering/maintaining relationships with veterinarians/producers, and (j) limitations to relationship development with veterinarians/producers. Within each of these themes, more descriptive subthemes were identified. The majority of veterinarians and producers share similar perspectives and opinions regarding the value of veterinarian-producer partnerships, key relationship characteristics, and limitations to relationship development. The findings presented represent the collective view of both populations and the 10 main themes aggregated into five main ideas.

### 4.1 Time as a barrier

Responses provided by both veterinarians and producers indicate time as a significant barrier to relationship development and maintenance. Veterinarians and producers both recognize that veterinarians navigate heavy caseloads that often exceed standard workday hours. This results in limited time available to meet producers’ needs and expectations. While fostering relationships results in additional clients, it also increases workload, leaving less time for themselves, their family, and their existing practice. Veterinarians view their lack of time as a key reason for not fostering and maintaining relationships with producers. This is supported by the overall lower percentage of veterinarians willing to partner with producers in areas of healthcare (72% versus 90%), goal achievement (67% versus 80%), and business expansion (49% versus 70%). Producers also find that time is a significant barrier to both relationship development and receiving animal care. Many producers are aware of veterinarians’ busy schedules, yet frustrated that animal care is often not available in a timely manner. Whether it is the time required for a veterinarian to make a farm call, or the time required to transport an animal to the clinic, time constraints have become a key frustration and barrier to relationship development. Both veterinarians and producers recognize that time will remain a barrier and see limited solutions to combat this challenge.

### 4.2 Business as a profit or burden

Across all interviews, the financial challenges associated with running a veterinary practice or a livestock operation were discussed. Every veterinarian addressed the financial burden and responsibility of caring for animals. Several veterinarians also spoke to the limited profit found in their practice. They described the burden of paying off debts while simultaneously charging reasonable fees to balance affordability with profitability. Many veterinarians have found limited profit in operating exclusively large animal practices. In fact, veterinarians’ willingness to partner with producers to grow their business rated lowest at 49% of all partnership willingness areas. Both veterinarians and producers feel the pressure of maintaining their operations/practices amid rising overhead and animal care costs. Veterinarians also recognize the challenge that producers face when making decisions related to animal care, as they too must make economic decisions when determining the types of services offered. For example, they must operate mixed animal practices, even if they prefer to practice exclusively on large animals, to ensure their financial stability. However, many veterinarians interviewed held the perspective that producers will not seek veterinary care if they believe the expense outweighs the animal’s market value. Veterinarians note financial decisions as the reason many producers provide their own animal care, regardless of whether they have the knowledge and skills to do so. As such, veterinarians perceive that producers negatively view their services, except in the case of emergencies, because regular veterinary involvement limits profit. Quantitative evidence does not support this perception as almost half, 48%, of producers seek veterinarian input for animal health questions and more than 60% expressed interest in animal health continuing education topics. Every producer stated that their animals are their livelihood. Therefore, if the cost of veterinary care is unaffordable, they will provide their own care and redistribute those funds into other parts of the operation. Although producers desire veterinary assistance whenever their animals need care, they lack the financial security to always obtain it in a traditional manner. They would like support from their veterinarian, in the form of education and training, to provide some services on their own. This would allow them to use funds conservatively; improving the business sustainability of their operation.

### 4.3 Communication is key

Veterinarians and producers agree that successful relationships are founded on clear and consistent communication. Their opinions differ regarding the medium through which communication happens. While many producers are interested in communicating across different mediums—social media, email, etc., veterinarians are more hesitant to pursue those forms of communication. The extremely low percentage (0–6.4%) of producers aware of veterinarians’ full range of services demonstrates this hesitancy in embracing various communication outlets. Producers expressed interest in telemedicine as a solution to the barriers of time and distance. However, many veterinarians were disinterested in telemedicine preferring in-person care. Overall, veterinarians recognize communication as key to relationships, yet they feel challenged to be consistent communicators because of time constraints. The veterinarian’s heavy workload limits their ability to connect with producers or respond promptly to their questions. This leads to the perception that they are unwilling to help. This challenge is coupled with some producers’ expectations that veterinarians should have 24/7 availability. While producers understand the busy nature of veterinarians’ work, there seems to be an underlying frustration that accessibility is not always possible. This lack of access and untimely communication has led some producers to believe their needs are not a priority. They feel they are better off providing care themselves because the delay in connecting with a veterinarian could adversely impact the animal’s health and thus the producers’ entire operation.

### 4.4 Competing perspectives

Review of producer and veterinarian feedback reveals a shared acceptance that barriers exist between these two groups, fueled by each believing a false narrative of the other. These misconceptions have resulted in relationships either built out of necessity or never built at all. Many veterinarians perceive that producers do not recognize the value of their services. Instead, they feel like a safety net for producers when care goes wrong. Further, they think that producers are strictly concerned with the cost of care as it compares to the animal’s market value. When having to choose between providing care or making a profit, they always decide in favor of profitability. Alternatively, many producers feel that veterinarians are less willing to help due to their limited accessibility and divisive attitude regarding producers providing animal care. Several producers were candid in their responses about the difficulties in making hard financial decisions to ensure operation sustainability while providing necessary animal health care. Producers feel they should be supported in providing some forms of animal healthcare themselves. Overall, many producers feel that veterinarians do not respect the choices they make regarding their operations, resulting in limited trust and a hesitation to form partnerships. Despite these unfavorable perceptions, veterinarians and producers actually share much common ground. While facing similar challenges to ensuring the success of their businesses—expense, weather, and availability of care—they both recognize the importance (74% of producers) and impact (56% of veterinarians) of continuing education, and the majority (> 65%) are willing to develop partnerships.

### 4.5 Mutual respect

Across all interviews, producers and veterinarians expressed a desire to create partnerships founded on mutual respect for decisions surrounding each other’s practices/operations. Producers respect veterinarians’ expertise. Forty-eight percent seek animal health care information from their veterinarian. Therefore, veterinarians have some influence and responsibility in building relationships. Communicating clearly and empathetically about challenges and seeking insight, then actively listening, to factors determining producers’ decisions may foster this respect.

## 5 Conclusion

Greater than 65% of participants indicated a desire to create partnerships for animal health and to achieve goals—a tacit acknowledgment by both parties of the importance of veterinarian/producer relationships. Additionally, more than 60% of producers expressed a high level of interest in participating in continuing education. Veterinarians, concurrently, expressed a need for more animal health education among producers and believe this effort will positively impact the veterinarian-producer relationship. Both parties believe in-person learning communities are the most effective means to gain knowledge and skills. Based on this information, we propose that collaboratively led applied education programs possess significant potential for developing partnerships by addressing the barriers of communication, perspective, respect, profit, and time. In this learning environment, veterinarians and producers have a “place at the table” promoting communication, mutual respect, and opportunities to share perspectives on goals, motivations, and experiences. Qualitative interview data suggests that veterinarians and producers desire these relationship characteristics and believe such traits can improve their practices/operations. The profit barrier could also be addressed through education by utilizing data to demonstrate the realized cost–benefit of preventive versus reactive animal health management. Time remains a serious hurdle in implementing educational programs and developing veterinarian/producer partnerships. Additional studies are needed to determine how to influence veterinarians and producers to prioritize educational programs and partnerships. One solution could be enlisting the support of extension programs, veterinary schools, and professional organizations to promote, incentivize, and implement these programs. Veterinarian and producer partnerships are the cornerstone of sustainable and profitable rural practices and small to medium-sized livestock operations. Collaborative education programs can provide the framework to remove existing partnership barriers and build a foundation for these relationships to grow and evolve.

## Data Availability

The datasets presented in this study can be found in online repositories. The names of the repository/repositories and accession number(s) can be found at: https://hdl.handle.net/1969.1/203139.
