# Supplementary material for: Education necessity for veterinary-producer relationship creation and sustainability: a mixed method study
Source: Front Vet Sci. 2025 Apr 17;12:1521440. doi: 10.3389/fvets.2025.1521440 (PMC12045065; doi:10.3389/fvets.2025.1521440)
Supplement: Supplementary file 1 [file Supplementary_file_1.DOCX]

**Appendix A**

**Veterinarian and Producer Assessments**

This appendix provides the open and closed-question surveys that veterinarian and producer participants completed to explore the willingness and barriers to producers and veterinarians creating partnerships to enhance the profitability and sustainability of practices/operations.


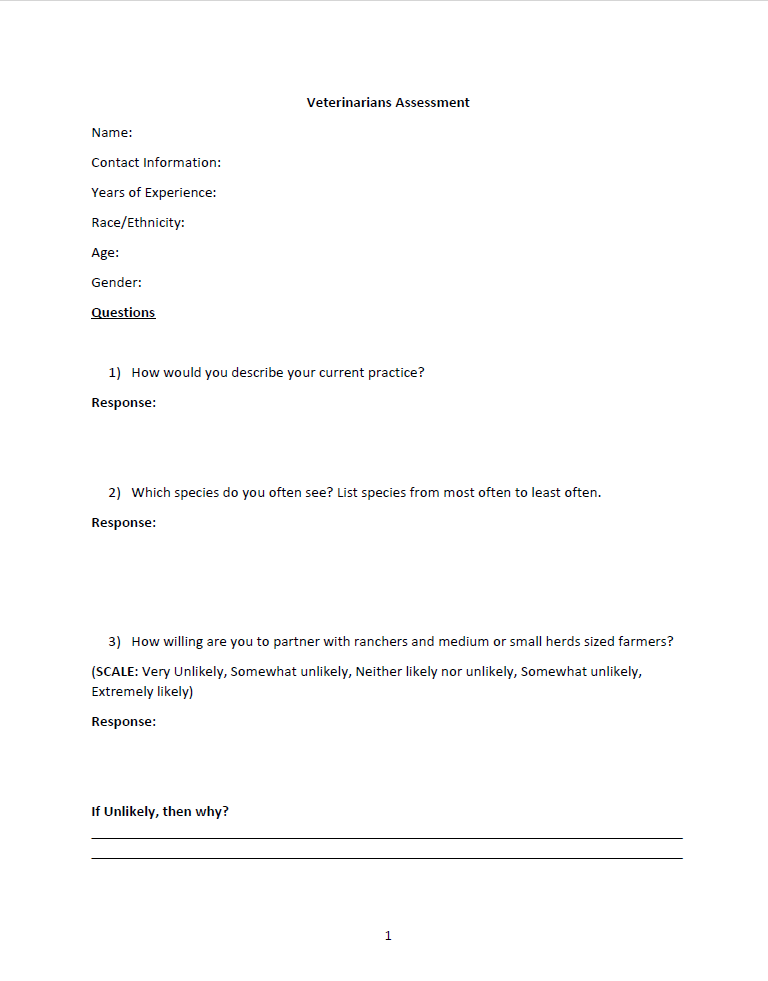


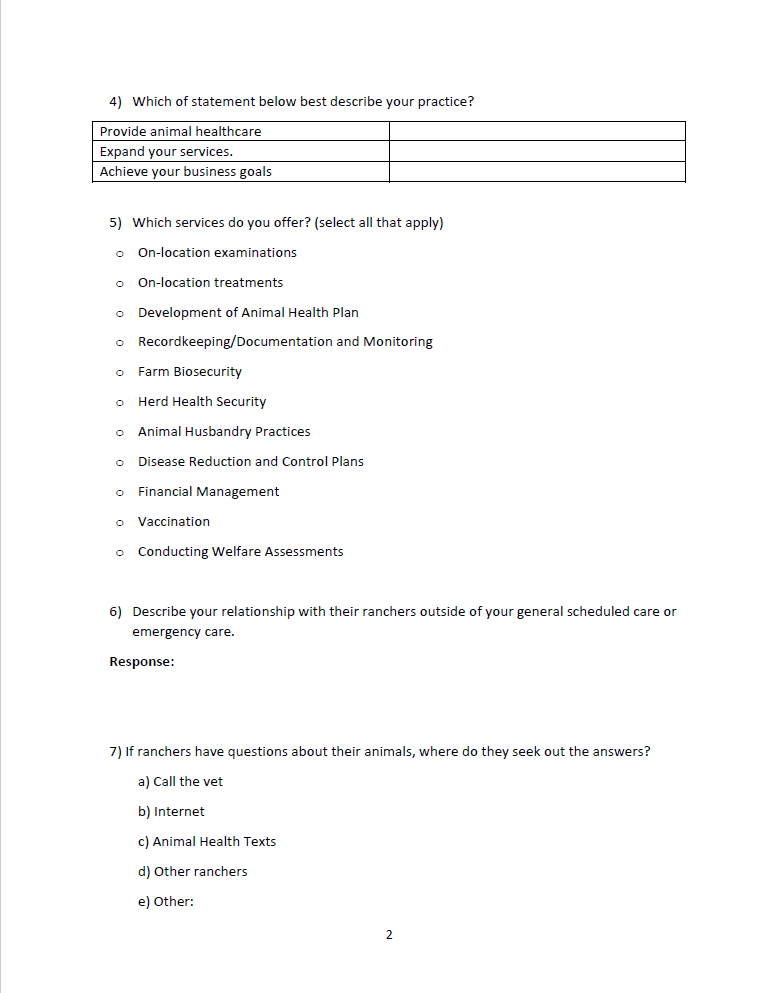


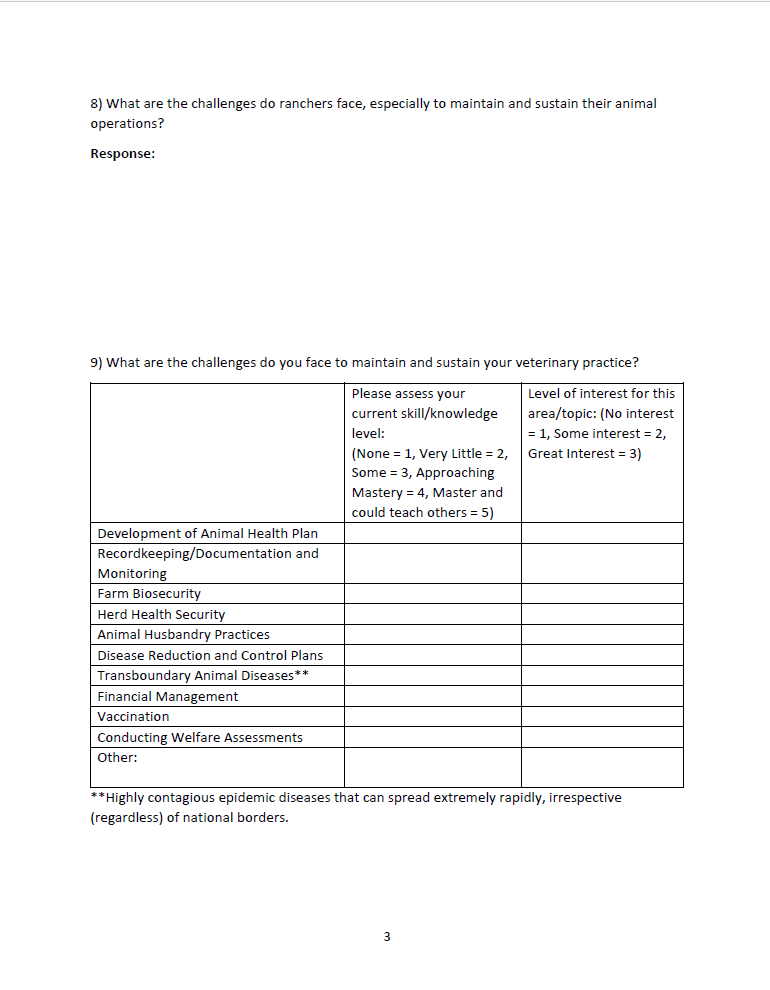


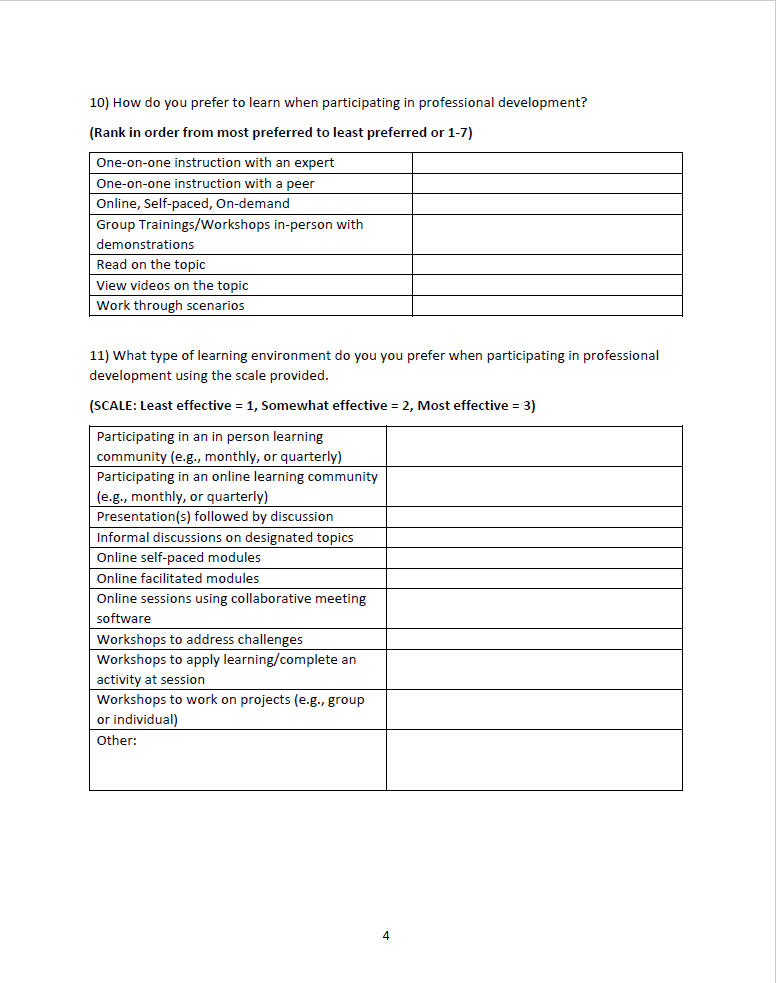


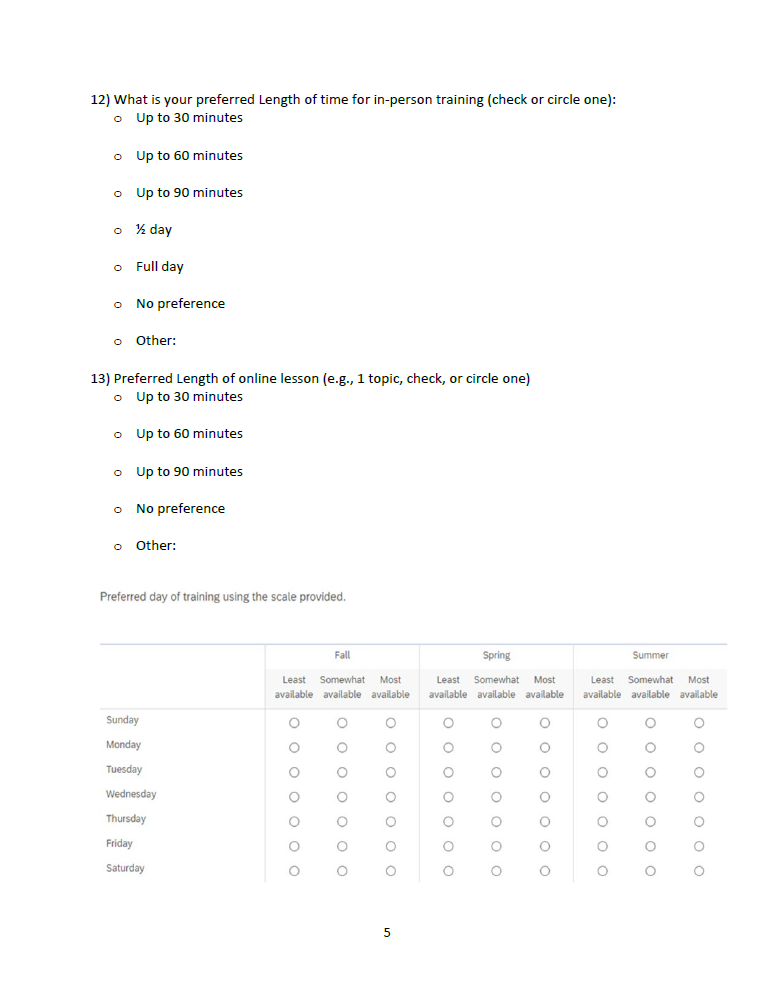


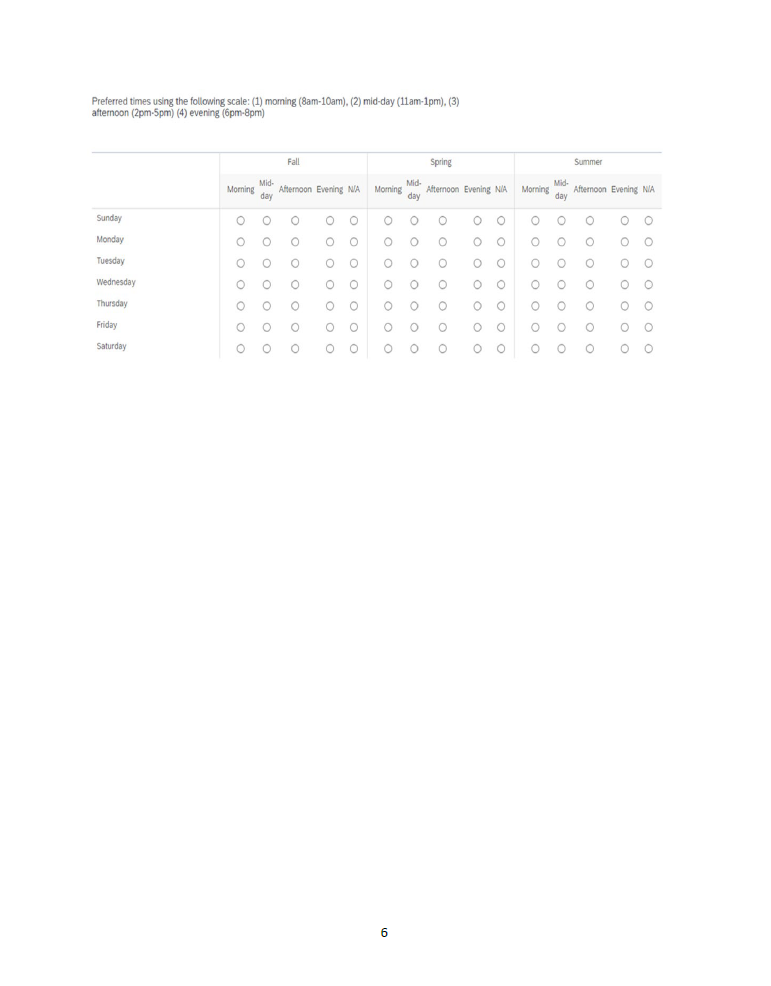


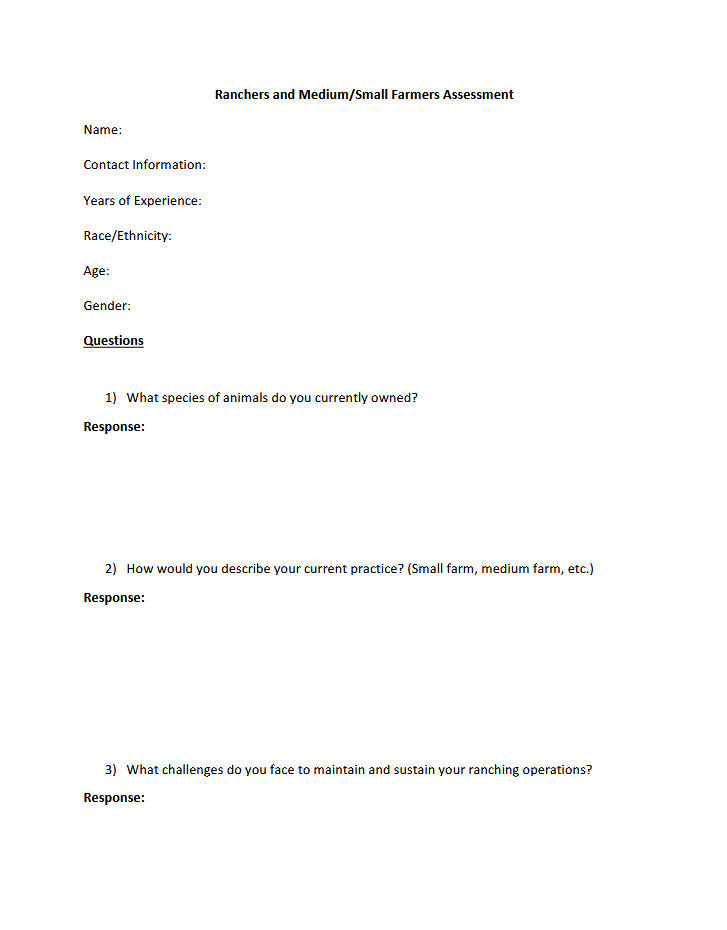


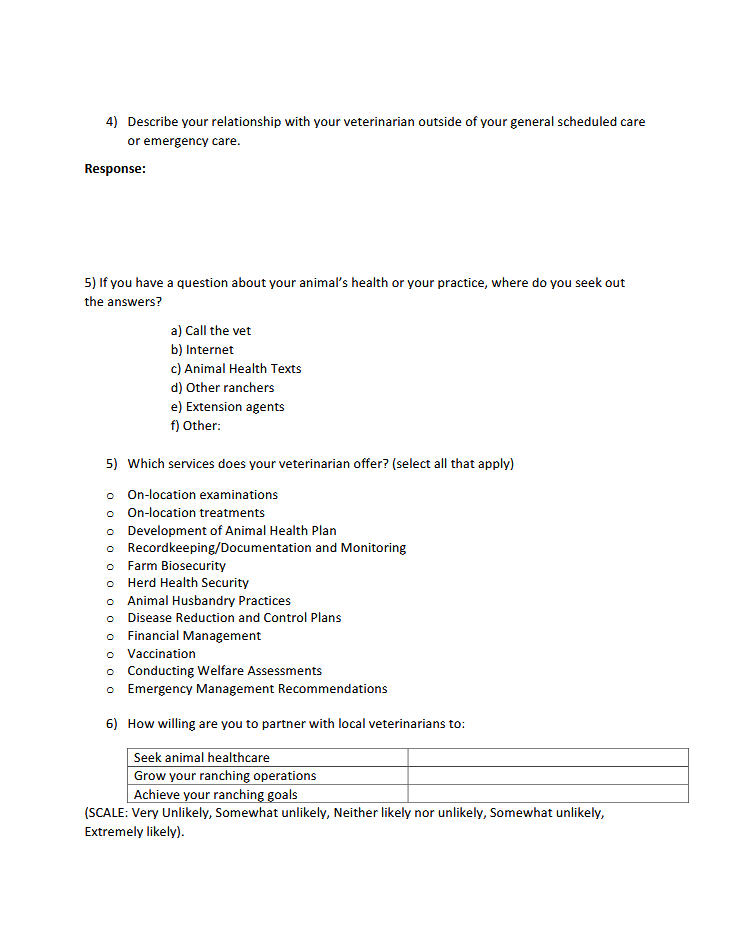


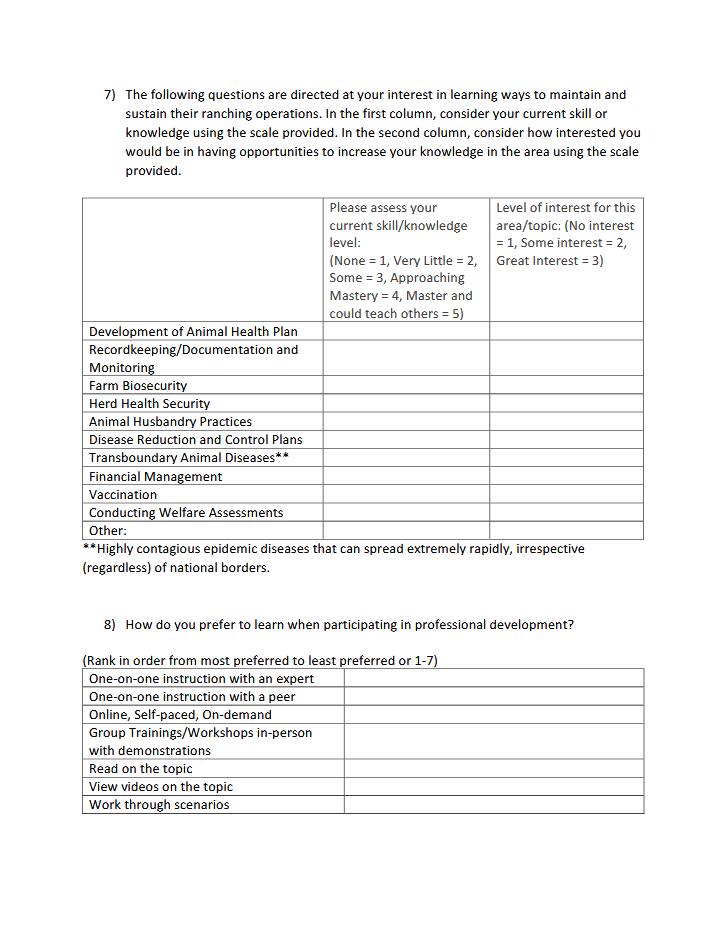


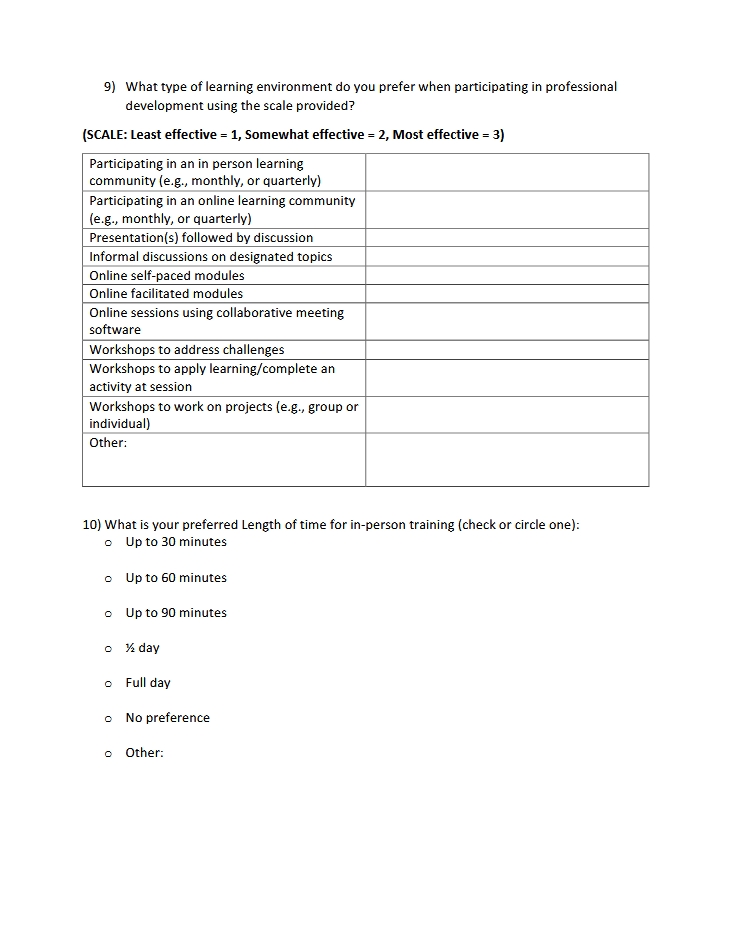


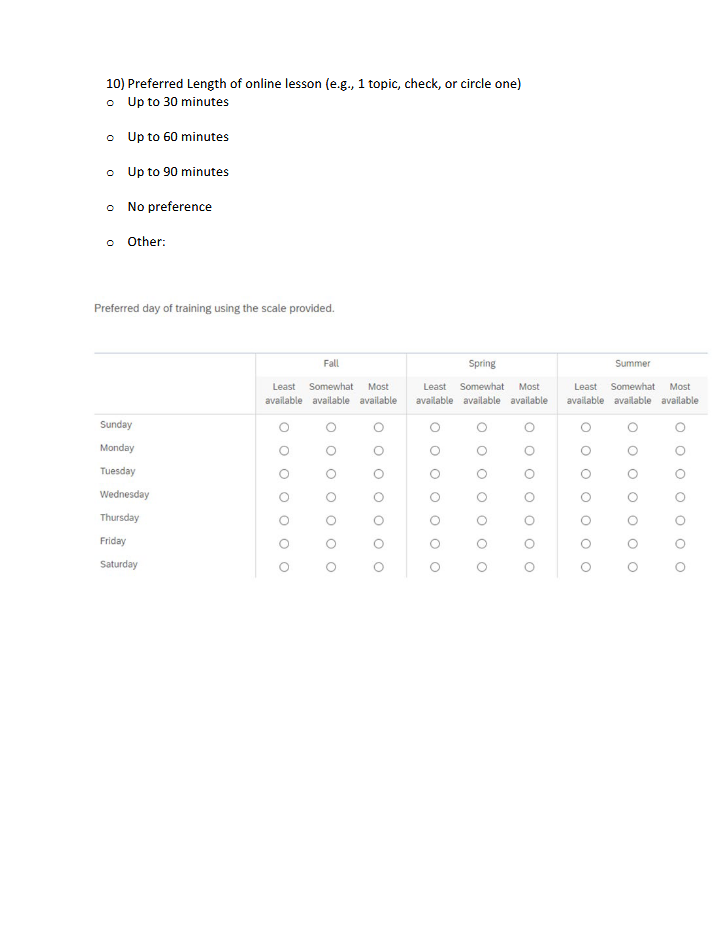


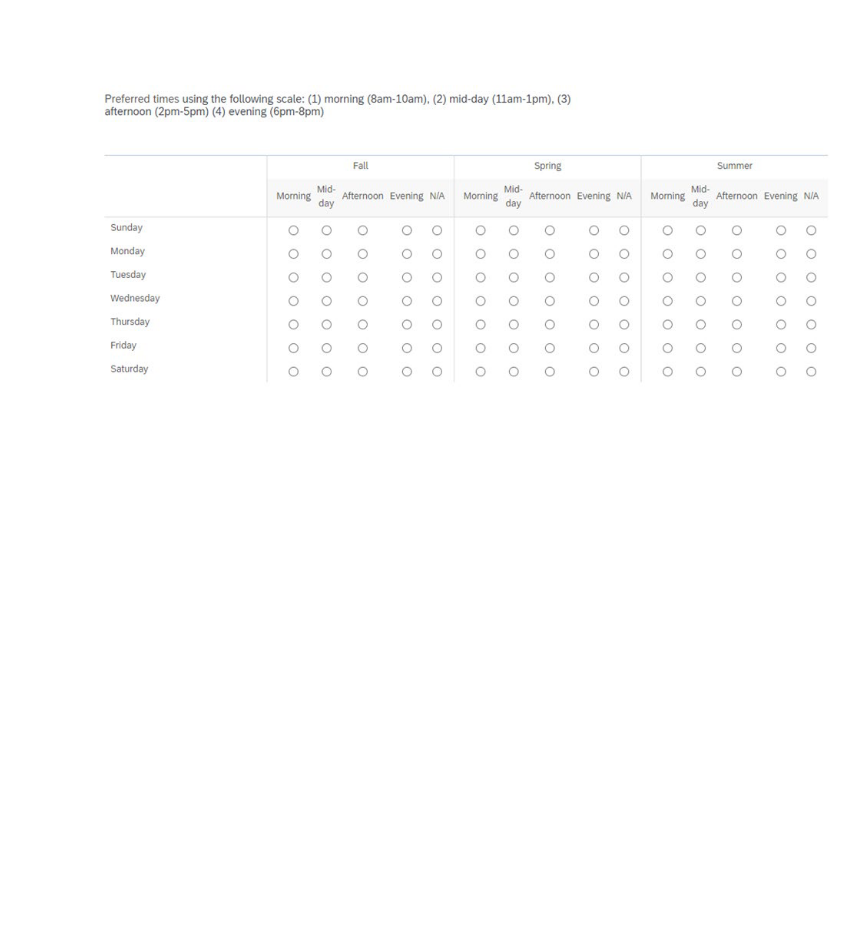


**Appendix B**

**Participant Follow-up Interview**

This appendix provides the follow-up interview questions that veterinarian and producer participants completed to elaborate on the willingness of and barriers to producers and veterinarians creating partnerships to enhance the profitability and sustainability of practices/operations.


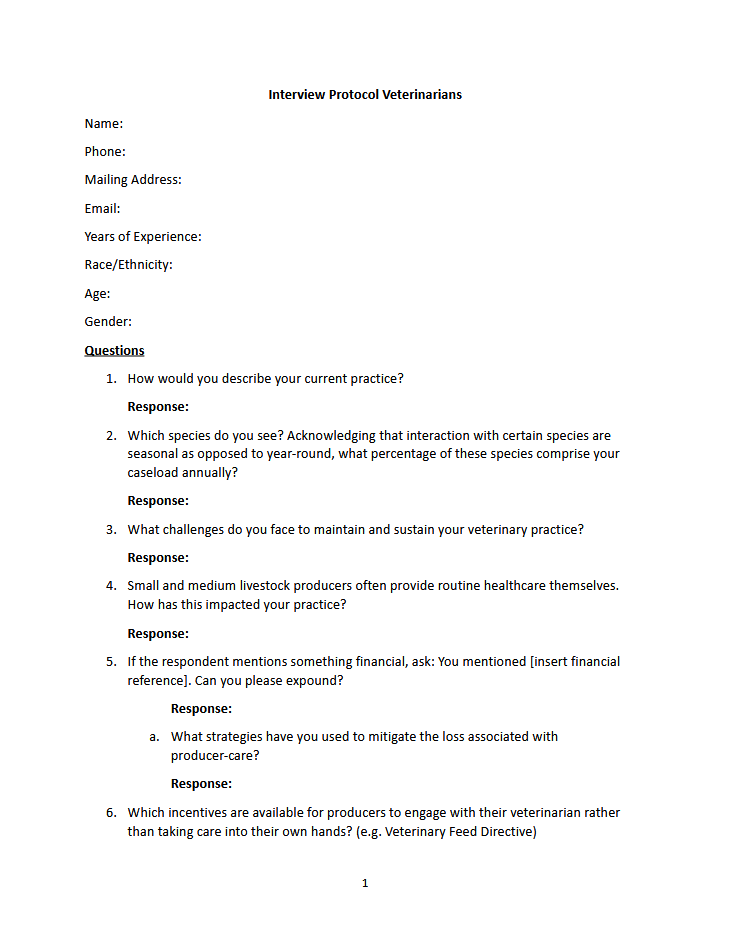


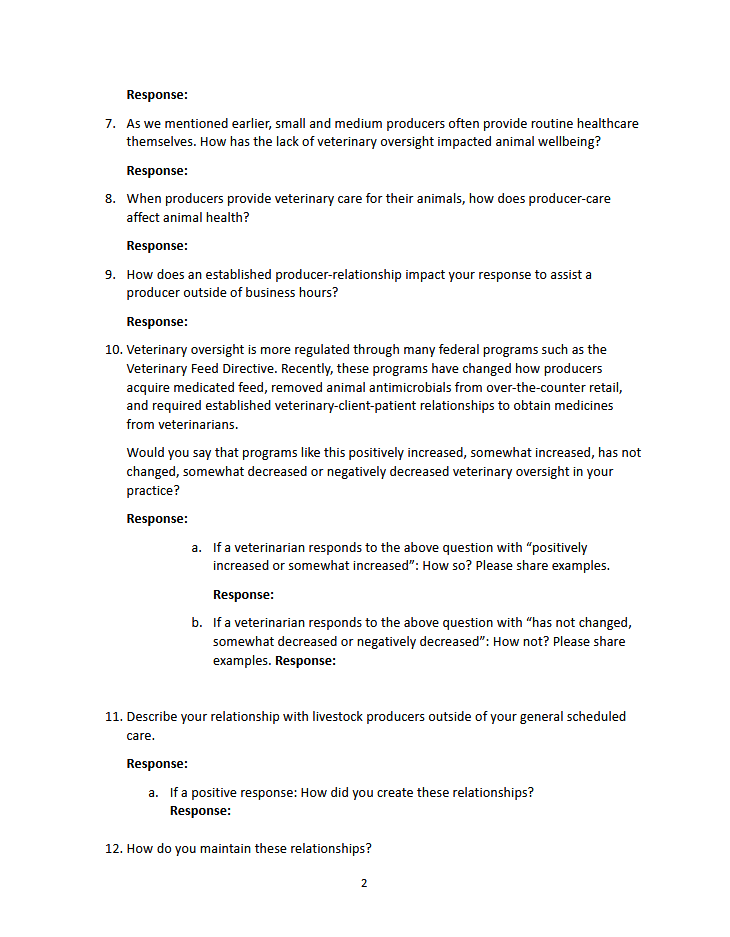


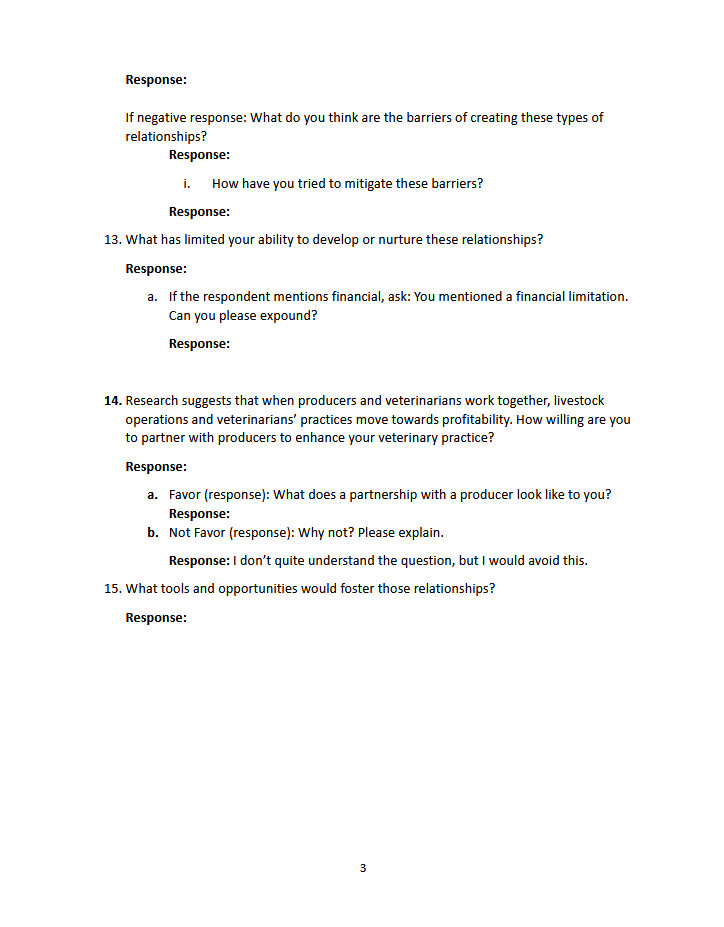


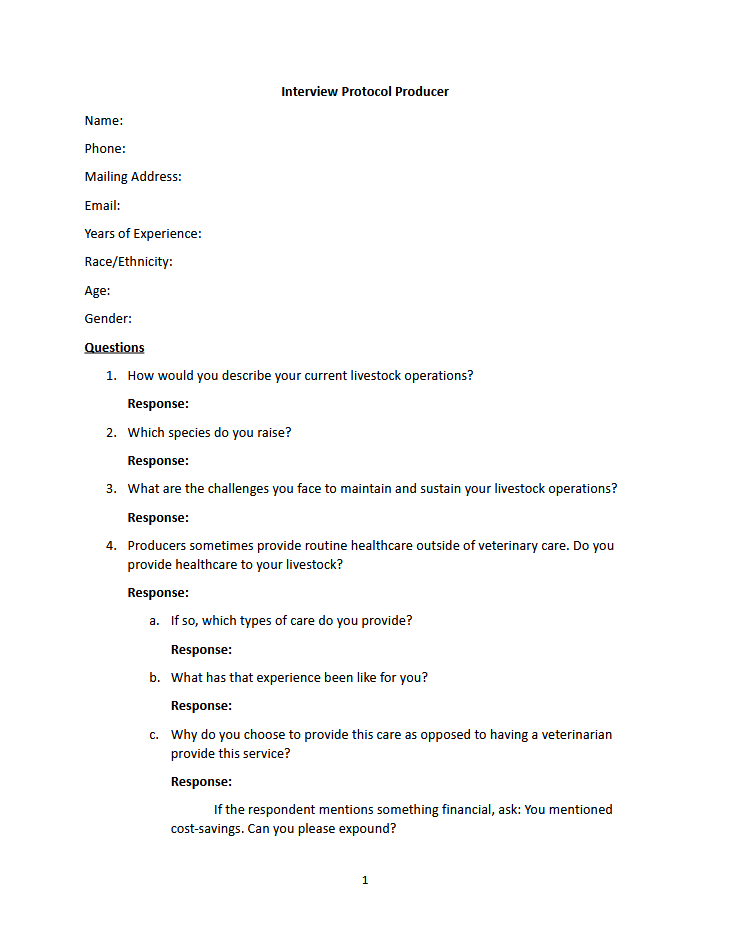


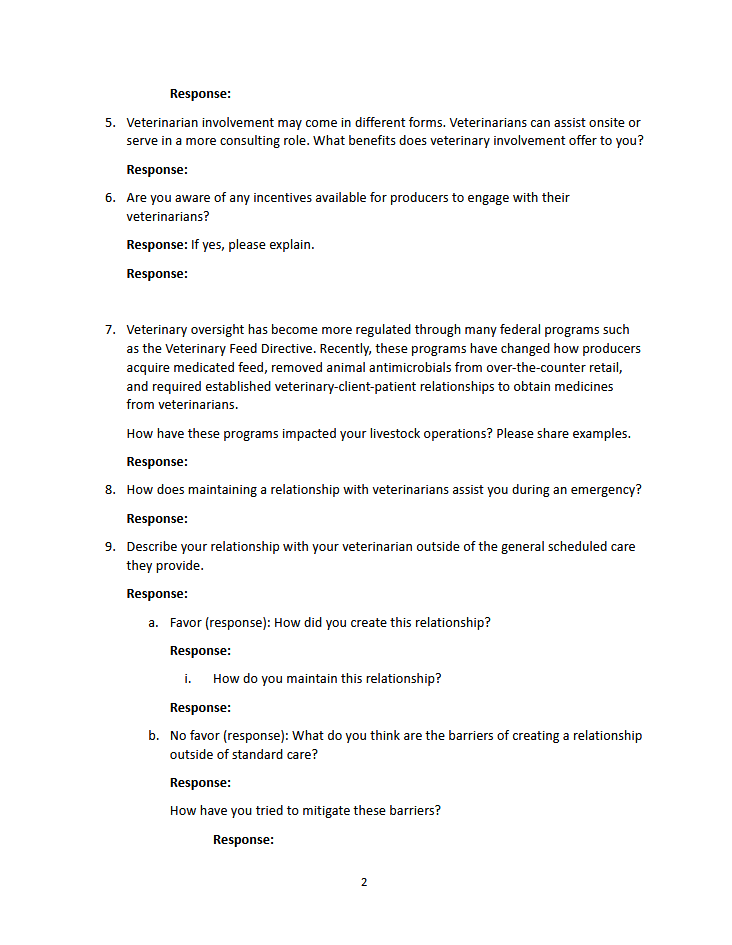


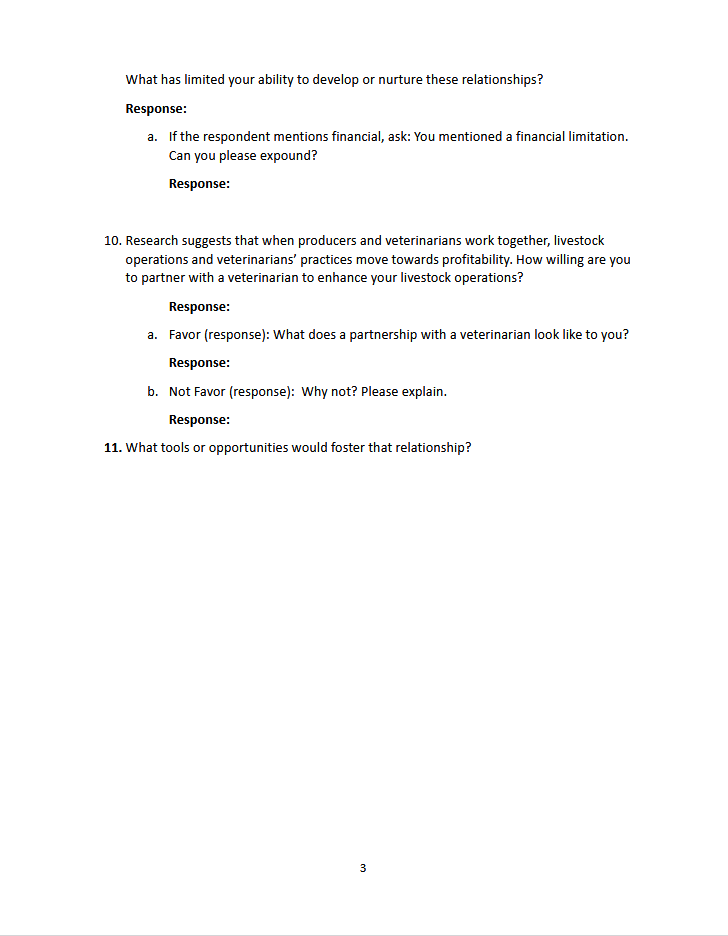


**Appendix C**

**Veterinarian Interview Response Themes and Subthemes**

This appendix categorizes the veterinarians’ responses to 12 interview questions into themes and subthemes.

**Theme 1: Practice Description**

The responses revealed twelve sub themes regarding veterinarians’ practice: (a) mixed animal, (b) primarily large animal, (c) primarily small animal, (d) specialty – seasonal, (e) bovine, (f) canine, (g) equine, (h), feline, (i) goat, (j) ovine, (k) poultry/avian, and (l) swine.

**Theme 2: Practice Sustainability Challenges**

The theme of “Practice Sustainability Challenges,” resulted in the following seven subthemes: (a) cost of care/maintenance, (b) economics of veterinary medicine, (c) facilities, (d) inefficiencies in practice and standards, (e) quality staffing, and (f) limited-service offerings.

**Theme 3: Barriers to Providing Veterinary Care**

Highlighting the theme of barriers to providing veterinary care seven sub themes emerged: (a) financial burden, (b) instant gratification, (c) limited profit, (d) no relationship – potential for delayed care, (e) provide care by self, (f) alternate sources for animal health care information, and (g) time.

**Theme 4: Incentives to Seeking Veterinary Care**

The theme of Incentives to Seeking Veterinary Care, resulted in the following four subthemes: (a) assistance in securing needs if a veterinarian is on record, (b) few incentives, (c) access to medicine and scripts, and (d) no incentives.

**Theme 5: Federal Programs**

The line of inquiry related to Federal Programs served as the foundation for seven subthemes: (a) positively increased, (b) negative impact, (c) has not changed, (d) feed control, (e) telemedicine, and (f) positive and negative practices continue.

**Theme 6: Producer Provided Animal Care**

Questions related to producer-provided animal care resulted in eight sub themes including (a) care products easily accessible, (b) limited recordkeeping for animal care, (c) no effect on practice, (d) request for telemedicine, (e) calls for care as last effort, (f) negative impact for animal and producer, (g) no impact on animal health, and (h) varied producer knowledge and skill set.

**Theme 7: Strategies to Mitigate Impact of Producer Provided Animal Care on Practice**

Responses to the question about strategies to mitigate impact led to four subthemes: (a) quality response/service, (b) client education, (c) increased communication, (d) seek and provide resources/solutions.

**Theme 8: Partnerships with Producers**

Two questions about producer partnerships led to the development of seven subthemes: (a) consistent communication, (b) intimate knowledge of care needs, (c) offer programming and education, (d) preference for in-person relationship, (e) producers as community, (f) support producers delivering animal care, and (g) no relationship – potential for delayed care.

**Theme 9: Methods for Fostering/Maintaining Relationships with Producers**

Responses to questions on how veterinarians foster and maintain relationships resulted in subthemes: (a) building trust, (b) consistent scheduled care, (c) hosting seminars/trainings, (d) increased collaboration, and (e) communication across mediums.

**Theme 10: Limitations to Relationship Development with Producers**

Questions regarding limitations to developing relationships resulted in seven subthemes: (a) lack of knowledge, (b) lack of time, (c) late communication, (d) unrealistic expectations, (e) individual capacity, (f) financial burden, and (g) limited profit.

**Appendix D**

**Producer Interview Response Themes and Subthemes**

This appendix categorizes the producers’ responses to 12 interview questions into themes and subthemes.

**Theme 1: Operation Description**

Through the responses provided related to operation description, ten sub themes emerged: (a) large operation, (b) medium operation, (c) small operation, (d) next generation, (e) bovine, (f) equine, (g) goat, (h) ovine, (ki) poultry/avian, and (j) swine.

**Theme 2: Operation Sustainability Challenges**

The theme of “Operation Sustainability Challenges,” resulted in five sub themes: (a) cost/availability of care, (b) cost of operation/overhead, (c) quality staffing, (d) risks to operation, and (e) transportation.

**Theme 3: Barriers to Seeking Veterinary Care**

There were ten subthemes highlighting barriers to seeking veterinary care: (a) financial burden, (b) copy previous generation practices, (c) limited profit, (d) no relationship – potential for delayed care, (e) preference for providing own care, and (f) time.

**Theme 4: Incentives to Seeking Veterinary Care**

The theme of Incentives to Seeking Veterinary Care, resulted in five subthemes: (a) assistance in securing needs if a veterinarian is on record, (b) access to more knowledge, (c) access to on-site care, (d) no incentives, and (e) consulting.

**Theme 5: Federal Programs**

Producers were asked how federal programs have impacted their livestock operations which served as the foundation for one subtheme: has not changed.

**Theme 6: Producer Provided Animal Care**

Questions related to producer-provided animal care revealed four subthemes: (a) call for care as last effort, (b) challenging, (c) preventative care is best, and (d) self-care is easy and sustainable.

**Theme 7: Partnerships with Veterinarians**

Three questions related to veterinarian partnerships led to the development of nine subthemes: (a) consistent communication, (b) intimate knowledge of care needs, (c) offer programming and education, (d) limited relationship outside of care, (e) veterinarians as community, (f) provide necessary care, (g) no relationship, (h) mutual respect, and (i) value cost of veterinarians’ services.

**Theme 8: Methods for Fostering/Maintaining Relationships with Veterinarians**

Responses to interview questions related to creating and maintaining veterinarian relationships resulted in five sub themes emerged: (a) building trust, (b) consistent scheduled care, (c) hosting seminars/trainings, (d) increased collaboration, and (e) communication across mediums.

**Theme 9: Limitations to Relationship Development with Veterinarians**

The question related limitations in veterinarian relationship development resulted in the emergence of only one subtheme: (a) lack of time.
